# The Clinical Relevance of Selected Cytokines in Newly Diagnosed Multiple Myeloma Patients

**DOI:** 10.3390/biomedicines11113012

**Published:** 2023-11-09

**Authors:** Michał Mielnik, Aneta Szudy-Szczyrek, Iwona Homa-Mlak, Radosław Mlak, Martyna Podgajna-Mielnik, Aneta Gorący, Teresa Małecka-Massalska, Marek Hus

**Affiliations:** 1 Department of Hematooncology and Bone Marrow Transplantation, Medical University of Lublin, 20-081 Lublin, Poland; 2 Department of Human Physiology, Medical University of Lublin, 20-080 Lublin, Poland; iwona.homa-mlak@umlub.pl (I.H.-M.);; 3 Department of Laboratory Diagnostics, Medical University of Lublin, Doktora Witolda Chodźki 1 Str., 20-093 Lublin, Poland; radoslawmlak@umlub.pl; 4 Department of Hematology and Bone Marrow Transplantation, Saint Jan of Dukla Oncology Centre of the Lublin Region, Doktora Kazimierza Jaczewskiego 7 Str., 20-090 Lublin, Poland

**Keywords:** multiple myeloma, angiogenesis, interleukins, VEGF, cytokines, cancer, tumor progression

## Abstract

Multiple myeloma (MM) is the second most common hematological neoplasm. Cytokines, chemokines, and their receptors, induced by the microenvironment of MM, participate in tumor growth, the attraction of leukocytes, cell homing, and bone destruction. This study aimed to assess the correlation between the pretreatment serum concentrations of interleukin-6 (IL-6), interleukin-8 (IL-8), angiogenic chemokine monocyte chemoattractant protein-1 (MCP-1), and vascular endothelial growth factor (VEGF) and the clinical outcomes and survival of patients newly diagnosed with MM. The study group consisted of 82 individuals. The IL-8 concentration was significantly positively correlated with the age of onset (*p* = 0.007), the International Staging System (ISS) stage (*p* = 0.03), the Eastern Cooperative Oncology Group (ECOG) performance status (*p* < 0.001), the degree of anemia before treatment (*p* < 0.0001), the degree of kidney disease (*p* < 0.001), and VEGF (*p* = 0.0364). Chemotherapy responders had significantly lower concentrations of IL-8 (*p* < 0.001), IL-6 (*p* < 0.001), and VEGF (*p* = 0.04) compared with non-responders. Patients with treatment-induced polyneuropathy had significantly higher levels of IL-8 (*p* = 0.033). Patients with a high level of IL-6 had a 2-fold higher risk of progression-free survival (PFS) reduction (17 vs. 35 months; HR = 1.89; *p* = 0.0078), and a more than 2.5-fold higher risk of overall survival (OS) reduction (28 vs. 78 months; HR = 2.62; *p* < 0.001). High levels of IL-6, IL-8, and VEGF demonstrated significant predictive values for some clinical conditions or outcomes of newly diagnosed MM patients. Patients with an early response to chemotherapy had a significantly lower concentration of these cytokines. A high pretreatment IL-6 concentration was an independent negative prognostic marker for newly diagnosed MM patients.

## 1. Introduction

Multiple myeloma (MM) is the second most common hematological malignancy [1]. It is caused by the proliferation of plasma cells capable of producing functionally impaired monoclonal immunoglobulins [2].

The unpredictable biological behavior of MM cells reflects the complex interactions between plasma cells and the diverse components of the bone marrow microenvironment, which is composed of extracellular matrix proteins, bone marrow stromal cells, inflammatory cells, and microvessels [3]. These complex interactions are pivotal in driving myeloma cell proliferation, angiogenesis, osteoclastogenesis, drug resistance, and, ultimately, disease progression [4]. The evolving landscape of MM treatment, with the introduction of novel agents, has underscored the need to elucidate the unique characteristics of myeloma cells, including their drug resistance mechanisms and their impact on the immune and non-immune microenvironment [5,6].

The role of pro-inflammatory proteins in the pathogenesis of MM is complex. Cytokines, with their dual involvement in pro-inflammatory and anti-inflammatory processes, have garnered attention in the context of cancer development [4]. They are unequivocally associated with inflammation, and an increase in the concentration of pro-inflammatory cytokines may promote tumor growth [7]. Tumors that produce few or no pro-inflammatory or anti-inflammatory cytokines exhibit limited growth due to restricted inflammation and vascular responses. On the other hand, increased production of pro-inflammatory cytokines causes angiogenesis and thus promotes tumor development [3,8]. Among the multitude of biologically active factors in the MM microenvironment, cytokines and chemokines and their receptors have emerged as key players influencing tumor growth, immune cell recruitment, cell homing, and bone destruction [9,10].

However, despite the presence of numerous cytokines in the MM microenvironment, the clinical significance of selected cytokines remains relatively unexplored in the literature. In this study, we aimed to address this gap by investigating the clinical relevance of four specific cytokines: interleukin-6 (IL-6), interleukin-8 (IL-8), vascular endothelial growth factor (VEGF), and monocyte chemoattractant protein-1 (MCP-1). These cytokines were chosen due to their known presence in the MM microenvironment and their potential implications for clinical outcomes.

Interleukin-6 (IL-6) is a pleiotropic cytokine that may have pro- and anti-inflammatory effects, depending on the signaling pathway. IL-6 acts by stimulating the differentiation of B-lymphocytes as well as the proliferation and activation of T-lymphocytes and by controlling their responses in the acute phase of inflammation [4]. Many researchers have indicated that IL-6 could play an essential role in the development of MM, as an increased concentration of this cytokine has been observed in many patients with MM. It plays a role in promoting angiogenesis, which supports tumor growth [3,11]. It promotes the survival and proliferation of MM cells by activating the JAK-STAT signaling pathway, and consequently, it can take part in the development of drug resistance in MM [12].

Interleukin-8 (IL-8), also known as CXCL8, is one of the pro-inflammatory CXC chemokines [13]. It is essential in many physiological and pathophysiological processes [14]. Different types of cells, such as neutrophils, endothelial cells, macrophages, and various cancer cells, produce IL-8 [15]. It acts as a chemoattractant, drawing immune cells and inflammatory mediators to the tumor site. Neutrophils, T cells, monocytes, endothelial cells, and some cancer cells carry its receptors (CXCR1 and CXCR2) [10]. Researchers have suggested that IL-8 could be a factor in cancer progression, mainly through its ability to enhance angiogenesis. IL-8 signaling can stimulate the proliferation and migration of MM cells and contribute to disease progression [10,15,16]. Therefore, controlling IL-8 expression may serve as a potential tool when searching for new therapies to manage cancer growth and metastasis [15].

Vascular endothelial growth factor (VEGF) works in several ways. In addition to its vascular activity, it increases osteoclastic bone-resorbing activity. It also enhances the adhesion of natural killer (NK) cells to the tumor endothelium. It can also induce migration, parathyroid hormone (PTH)-dependent cAMP accumulation, and alkaline phosphatase in osteoblasts [17,18,19]. Stromal cells, endothelial cells, and osteoclasts secrete angiogenic cytokines, such as VEGF, thus promoting plasma cell growth, migration, and survival, as well as paracrine cytokine secretion and angiogenesis in the bone marrow. In MM, VEGF is produced by both MM cells and the stromal cells of the bone marrow [20,21].

Angiogenic chemokine monocyte chemoattractant protein-1 (MCP-1) is one of the CC chemokines secreted by MM cells. It acts as a potent chemoattractant for a subset of T lymphocytes, myeloma cells, monocytes, eosinophils, basophils, and endothelial cells through its CCR2 receptor [22,23]. Moreover, MCP-1 is the first CC chemokine observed to play a direct role in tumor angiogenesis. It enhances the inflammatory milieu of the MM microenvironment, fostering interactions between MM cells and immune cells [24]. MCP-1 has been associated with the development of osteolytic lesions, a common complication in MM [25].

Our study sought to establish the correlation between serum levels of these cytokines—IL-6, IL-8, MCP-1, and VEGF—and the clinical outcomes of newly diagnosed MM patients.

## 2. Materials and Methods

### 2.1. Study Group

This study included 82 consecutively enrolled patients who met the inclusion and exclusion criteria. These individuals were diagnosed based on the SLiM CRAB criteria [26]. The assessment of their disease stage was based on the Durie–Salomon criteria [27] and the International Staging System (ISS) classification [28]. Their performance status was determined by the Eastern Cooperative Oncology Group (ECOG)/World Health Organization (WHO) recommendations [29]. We included consecutive patients with MM diagnosed in our clinic during the recruitment period. Patients needed to be treatment-naïve and able to sign an informed consent form for the study. The obtained serum samples were required to be adequate to perform the preplanned tests. The exclusion criteria were a second active malignancy, previous systemic therapy for any malignancy shorter than two years before entering the study, autoimmunological diseases, and active infections.

The patients were treated with standard-of-care therapies and followed to obtain clinical data. Based on a thorough anamnesis, we recorded the type of the disease, the patients’ performance status, the stage of the disease at diagnosis, any genetic aberrations, and the degree of anemia before treatment, as these are recognized factors involved in the development and course of MM [30]. We also recorded any known exposure to chemical agents (such as fertilizers, paints, industrial cleaners, and other chemicals), weight loss before diagnosis, and smoking, as these have been reported as negative prognostic markers in numerous malignancies [31,32,33]. We classified the patients’ place of residence based on its population size (i.e., city ≥ 5000 inhabitants; village < 5000 inhabitants). We assessed the response to treatment according to the current International Myeloma Working Group (IMWG) guidelines [34]. In addition, we performed a detailed evaluation of the adverse effects of the administered chemotherapy (CTH) (hematological and non-hematological toxicities, including infections, polyneuropathies, thromboembolic events, diarrhea, and constipation, as well as other less frequent adverse events) in accordance with the Common Terminology Criteria for Adverse Events (CTCAE) v 5.0 [35]. When we specify kidney function as being “A” or “B” in this manuscript, this refers to a creatinine level of < or ≥2.0 mg/dL, respectively.

The control group consisted of 49 healthy individuals, matching the age and gender of the study group.

The studied material consisted of approximately 4.5 mL of peripheral blood collected into serum gel tubes before the start of treatment at the Department of Hematooncology and Bone Marrow Transplantation in Lublin (Poland) between 2015 and 2019. Follow-up included data from 2015 to 2021. The Committee of Ethics and Research at the Medical University of Lublin approved the study design (No. KE-0254/26/2015). Each patient provided written informed consent for the study after receiving an explanation of the planned form and scope of the use of data, the rules of processing and using the personal data collected during the study, and the rules and methods for conducting the study. Participation in the study was voluntary. The patients could revoke their consent at any time. This study was conducted in strict adherence to the principles of the Declaration of Helsinki.

### 2.2. Cytokine and Chemokine Measurements

Using flow cytometry with Cytometric Bead Array (CBA) solutions can detect a variety of soluble and intracellular proteins, including cytokines, chemokines, growth factors, and phosphorylated signaling proteins. Compared with the traditional western blot and ELISA techniques, this method can reduce the time to results and the sample requirements [36].

In this study, we used the BD CBA Human MCP-1 and VEGF Flex Set with a BD CBA Human Soluble Protein Master Buffer Kit. We also utilized the BD CBA Human IL-6 and IL-8 Enhanced Sensitivity Flex Set in conjunction with a BD CBA Human Enhanced Sensitivity Master Buffer Kit, a BD FACSCanto™ II flow cytometer, and FCAP Array™ Software version 3.0 (Becton Dickinson, Franklin Lakes, NJ, USA). The assays comprised a recombinant protein standard for internal control and a means to generate a standard curve and subsequent quantitative analysis. The diluted standards served as a basis to create a standard curve for each analyte covering defined concentrations (10 to 2500 pg/mL for the BD CBA Human Soluble Protein Flex Set and 274 to 200,000 fg/mL for the BD CBA Human Enhanced Sensitivity Flex Set). We determined the theoretical limits of detection for MCP-1 (1.3 pg/mL), VEGF (4.5 pg/mL), IL-6 (68.4 fg/mL), and IL-8 (69.9 fg/mL) by evaluating the estimated result for the average median fluorescence intensity (MFI) of the negative control (0 pg/mL or 0 fg/mL, *n* = 30 for each set) plus 2 standard deviations. We diluted the serum samples 1:4 before transferring them to the assay tubes to ensure that their median fluorescence values fell within the range of the generated standard curves.

As recommended by the manufacturer, we reconstituted and serially diluted the lyophilized BD CBA Human Flex Set standards directly before mixing them with the capture beads and the detection reagent. We ran the standards from least concentrated (0 pg/mL or 0 fg/mL) to most concentrated (Top Standard) to facilitate analysis via FCAP Array software version 3.0. Afterward, we diluted the capture beads to their optimal concentrations.

The Enhanced Sensitivity Detection Reagent (Part B) provided in the Human Enhanced Sensitivity Master Buffer Kit was lyophilized. Therefore, we rehydrated it and diluted it to its optimal concentration before usage. The BD CBA Human Enhanced Sensitivity Flex Set System uses a two-step detection system. First, we mixed the Human Detection Reagent (Part A) provided with each BD CBA Human Enhanced Sensitivity Flex Set with the other BD CBA Human Detection Reagents (Part A) and diluted this to the optimal volume (20 μL per test) before adding the Human Detection Reagents (Part A) to the assay tube.

The phycoerythrin (PE) detection reagent provided with the BD CBA Human Soluble Protein Flex Set was mixed with the other BD CBA Human Soluble Protein Flex Set PE detection reagents and diluted to the optimal volume per test (50 μL per test) before adding the PE detection reagents to a given tube or assay well.

Following the manufacturer’s instructions, we verified the instrument settings (i.e., compensation and voltages) before each experiment using the following beads: A9, PE-F1, F1, F9, and A1. Following the preparation and dilution of the individual assay components, we transferred the standards, samples, mixed capture beads, and mixed detection reagents to the appropriate assay tubes for incubation and analysis. After the sandwich complexes (capture bead + analyte + detection reagent) were incubated, we measured them using flow cytometry to identify particles with fluorescence characteristics of both the bead and the detector. The intensity of PE fluorescence of each sandwich complex revealed the concentration of that particular analyte. We acquired the samples on their preparation day to prevent the increased background and reduced sensitivity resulting from prolonged storage. We recorded at least 5000 events per analyte. After acquiring samples on a flow cytometer, we used FCAP Array™ software version 3.0 to generate the results in graphical and tabular formats. Supplementary Figure S1 shows a visual presentation of the tested cytokines’ concentrations.

### 2.3. Statistical Analysis

The computer programs MedCalc, version 15.8 PL, and Statistica, version 13 PL, were used for statistical analysis. We retrospectively calculated the sample size in the acquired data set. The post hoc calculation compared the percentages of patients with high or low IL-6 levels and 3-year survival as a primary endpoint. Most medical studies consider a *p*-value below 0.05 to reject the null hypothesis. Thus, a type I error (alpha) of 0.05 was used. For type II errors, we set a beta cut-off of 0.2 to achieve 80% statistical power. Considering that 78% of patients with a low level of IL-6 and 40% of patients with a high level of IL-6 achieved 3-year survival and considering the ratio of sample sizes in the compared groups (1:1), we estimated that at least 62 patients should be included in the study group. To ensure adequate statistical power, we further extended the study group by an additional 30%. Thus, we estimated that the minimal sample size for our study group was equal to 81. The continuous data distribution’s normality was assessed using the D’Agostino–Pearson test. Since the studied continuous variables were not normally distributed, we used the median for the concentration measurement and the interquartile range and the minimum–maximum range as the measures of dispersion.

For the same reason, where applicable, non-parametric tests were used. The evaluation of comparisons in terms of the concentration of tested cytokines depending on the demographic and clinical variables was performed using the Mann–Whitney U test. Moreover, we used Spearman’s rank correlation test to assess the continuous correlation between the tested cytokines and selected demographic and clinical variables. In cases of multiple comparisons, the Bonferroni correction was applied. We analyzed the impact of the studied variables on the reduction in time to progression (i.e., PFS) and OS using the log-rank test for univariate analysis and Cox’s logistic regression for multivariate models. All statistically significant results of the univariable analysis were considered potentially valuable for multivariable analysis. In cases where the composite (e.g., disease stage according to ISS) and its constituent variables (e.g., albumin or β2-microglobulin (B2M) levels) were statistically significant according to the univariable analysis, only the first one was included in the multivariate models.

Additionally, the backward elimination method revealed that only the ISS stage, “A/B” renal function, and 17p deletion were significantly related to PFS; thus, we used only these variables for adjustment in the multivariable analysis. For the same reason, only sex, auto-HSCT, and IL-6 were finally used for adjustment in the multivariable OS analysis. Finally, we generated survival curves using the Kaplan–Meier estimation method. Receiver operating characteristic (ROC) curves helped assess the tested cytokines’ diagnostic usefulness in differentiating between clinical conditions. For the analysis of the ROC curves, factors whose differentiation had a chance to be significant were selected based on the analysis of comparisons. Wherever this study’s results are reported as “high” or “low”, we refer to values above and below the median. OS was defined as the length of time from the date of diagnosis until death, whereas PFS was the length of time from the date of diagnosis to progression (as defined by the IMWG criteria) or death. Response to therapy was defined as achieving a minimal response (MR), partial response (PR), very good partial response (VGPR), complete response (CR), or stringent complete response (sCR) according to the IMWG criteria. On the contrary, no response was defined as the patient having stable disease (SD) or progressive disease (PD) during treatment.

**Figure 1:**
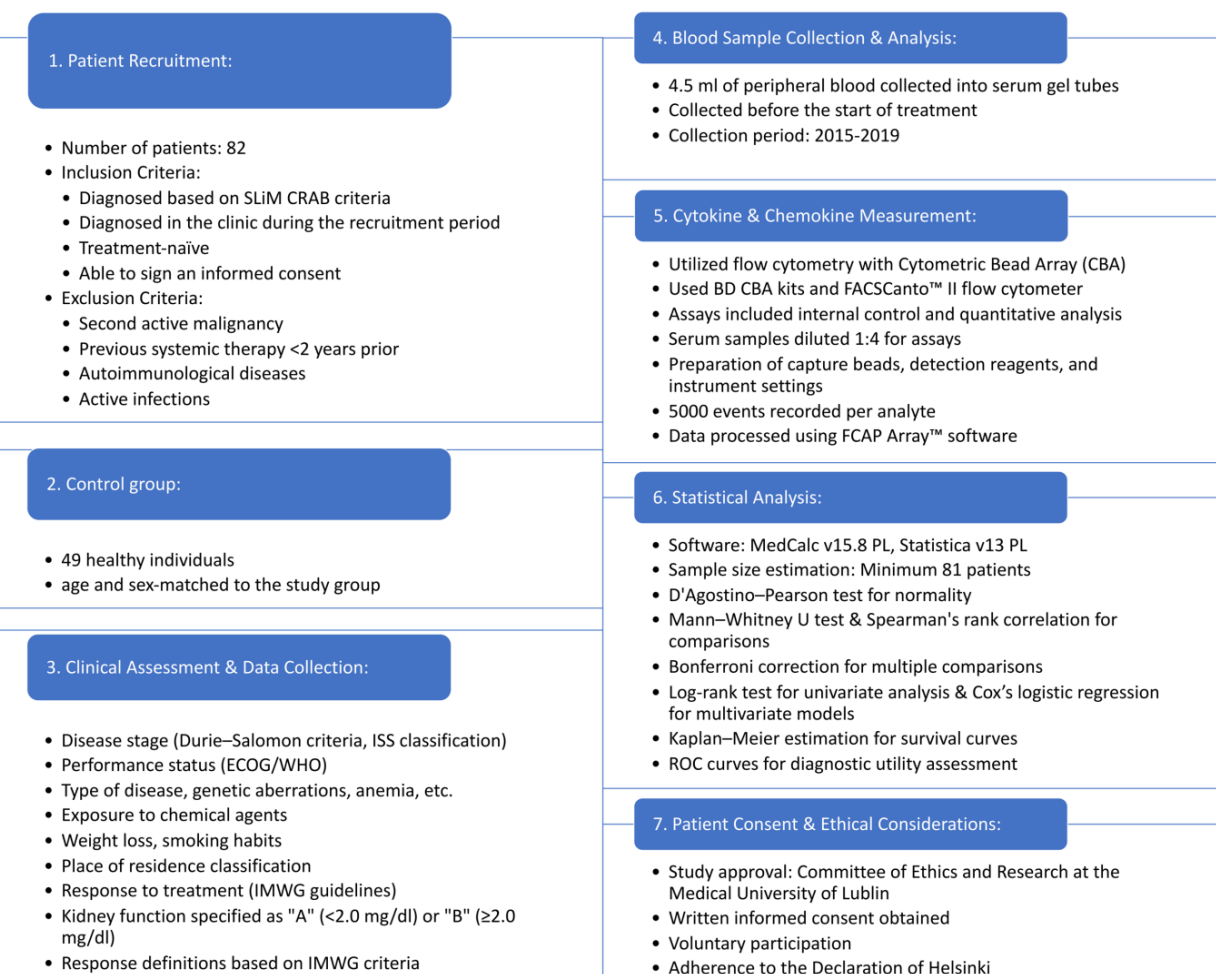
Schematic diagram of the study design.

Figure 1 shows a schematic diagram of the study design.

## 3. Results

### 3.1. Characteristics of the Study Group

The study group comprised 82 patients with MM (51.2% males). The median age in this group was 65 years (range: 37–87 years), with 51.2% over 65. MM with a monoclonal component (87.8%) was the most frequent diagnosis. According to the ISS, most patients were classified as stage 3, while 41.5% were in ECOG stage ≤ 1. All patients received CTH. Most received the thalidomide-based regimen (cyclophosphamide, thalidomide, dexamethasone (CTD); 46.3%), 35% received bortezomib-based regimens (bortezomib (cyclophosphamide) dexamethasone (V(C)D)/bortezomib, melphalan, prednisone (VMP)/bortezomib, adriamycin, dexamethasone (PAD)), and 17% received bortezomib, thalidomide, dexamethasone (VTD) regimens. In addition, 40.8% of the studied group underwent autologous hematopoietic stem cell transplantation (auto-HSCT). Table 1 presents the detailed demographic and clinical characteristics of the study group.

### 3.2. Comparison of Serum Concentrations of Tested Cytokines between the Study and Control Group

There was a significantly higher median IL-6 in the control group compared to the study group (3116.0 (interquartile range: 1793.7–4939.0) vs. 0.1 (interquartile range: 0.1 to 11,112.0), respectively; *p* = 0.0005; Supplementary Figure S2A). There was a significantly lower median IL-8 in the control group compared to the study group (4555.0 vs. 14,823.5, respectively; *p* < 0.0001; Supplementary Figure S2B). The control group had a significantly lower median VEGF compared to the study group (31.1 vs. 38.7, respectively; *p* = 0.0059; Supplementary Figure S2C). A significantly higher median MCP-1 was observed in the control group compared to the study group (228.3 vs. 161.6, respectively; *p* = 0.0001; Supplementary Figure S2D).

### 3.3. Comparison of Serum Concentrations of Tested Cytokines Depending on Demographic and Clinical Factors

Table 2 presents detailed data comparing the IL-6, IL-8, VEGF, and MCP-1 concentrations depending on the demographic and clinical variables.

Patients who lived in villages had a significantly higher serum concentration of IL-6 than those who resided in cities (median: 756.68 vs. 0.10 fg/mL; *p* = 0.047). People exposed to chemical or physical agents had significantly higher concentrations of IL-6 than individuals not exposed to such factors (median: 2389.75 vs. 0.10 fg/mL; *p* = 0.038). We demonstrated that in former smokers, the concentration of IL-6 was significantly higher than in those who never smoked (median: 3873.71 vs. 0.10 fg/mL; *p* = 0.032). Patients with stage 2 or 3 according to ISS had a higher concentration of IL-6 than those with stage 1 (median: 1049.94 vs. 0.10 fg/mL; *p* = 0.044). There were significantly higher concentrations of IL-6 in individuals with body-weight loss before treatment than in subjects whose body weight remained unchanged (median: 4076.92 vs. 0.10 fg/mL; *p* < 0.001).

In patients over 65, the concentration of IL-8 was significantly higher than in younger patients (median: 18,836.52 vs. 10,768.45 fg/mL; *p* = 0.004). Subjects in stage 2 or 3 according to ISS had a significantly higher concentration of IL-8 than those in stage 1 (median: 17,219.26 vs. 10,976.64 fg/mL; *p* = 0.031). Patients with kidney function assessed as “B” had a significantly higher concentration of IL-8 than subjects with kidney function assessed as “A” (median: 46,279.57 vs. 12,186.41 fg/mL, *p* < 0.001). We found that in individuals with performance status ≥ 1, the concentration of IL-8 was significantly higher than in those with an ECOG of 0 (median: 16,053.48 vs. 6870.38 fg/mL; *p* = 0.024). A significantly higher concentration of IL-8 was also found in people with pretreatment anemia than in patients with a normal hemoglobin concentration (median: 18,798.65 vs. 7229.50; *p* < 0.001).

The patients with kidney function assessed as “B” had significantly higher VEGF concentrations than those with kidney function assessed as “A” (median: 50.15 vs. 32.83 pg/mL; *p* = 0.004).

We did not observe a statistically significant association between the MCP-1 serum concentration and any demographic or clinical parameters.

### 3.4. Comparison of the Concentrations of the Tested Cytokines Depending on the Response to CTH

**Figure 2:**
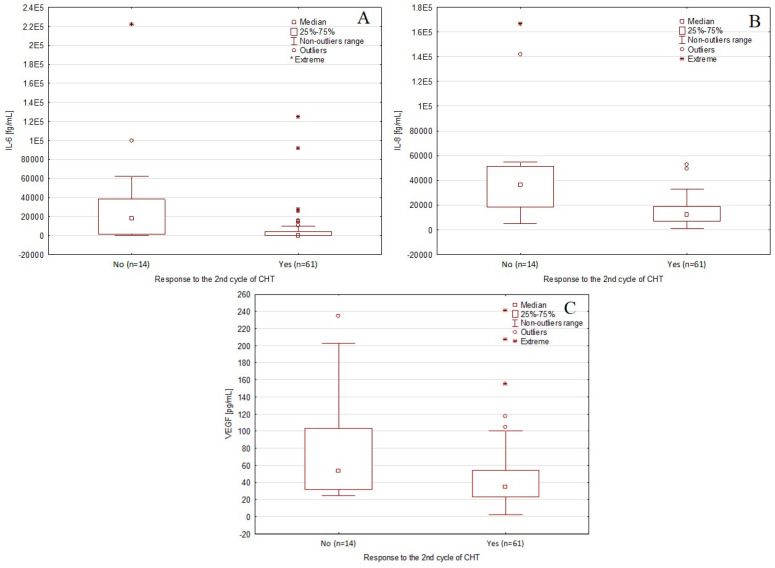
Box and whisker plots showing the comparisons of (**A**) IL-6, (**B**) IL-8, and (**C**) VEGF baseline serum concentrations depending on the occurrence of a response to the second cycle of chemotherapy. Abbreviations: CTH—chemotherapy; IL-6—interleukin-6; IL-8—interleukin-8; VEGF—vascular endothelial growth factor.

Patients who responded to receiving two cycles of CTH had significantly lower concentrations of IL-6 compared with non-responders (median: 0.10 vs. 17,777.91 fg/mL; *p* < 0.001; Figure 2A), significantly lower concentrations of IL-8 than non-responders (median: 12,096.70 vs. 36,113.32 fg/mL; *p* < 0.001; Figure 2B), and significantly lower VEGF concentrations than non-responders (median: 34.65 vs. 53.97 pg/mL; *p* = 0.040, Figure 2C).

However, we did not observe a statistically significant association between the tested cytokines concentrations and the response to CTH after the 4th, 6th, and 8th cycles (Supplementary Table S3).

Moreover, we did not observe a statistically significant association between the tested cytokines concentrations and achieving a response to CTH during the treatment period.

We did not observe a statistically significant association between the MCP-1 serum concentration and a response to CTH.

Detailed data comparing the IL-6, IL-8, VEGF, and MCP-1 concentrations depending on the response to CTH are presented in Table 3.

### 3.5. Correlations between the Selected Demographic, Clinical, and Laboratory Variables and the Tested Cytokines

We found a statistically significant positive correlation between IL-6 and IL-8 (moderate correlation; rho = 0.580; *p* < 0.001).

There was a statistically significant positive correlation between IL-8 and the age of onset (weak correlation; rho = 0.297; *p* = 0.007), worse renal function (moderate correlation; rho = 0.466; *p* <0.001), higher ISS stage (weak correlation; rho = 0.242; *p* = 0.030), higher ECOG performance status (moderate correlation; rho = 0.445; *p* < 0.001), degree of anemia before treatment (moderate correlation; rho = 0.520; *p* < 0.001), degree of kidney disease (weak correlation; rho = 0.396; *p* < 0.001), creatinine (weak correlation; rho = 0.321; *p* = 0.003), B2M (moderate correlation; rho = 0.415; *p* = 0.001), CRP (weak correlation; rho = 0.361; *p* = 0.001), on-treatment anemia (moderate correlation; rho = 0.372; *p* < 0.001), and VEGF (weak correlation; rho = 0.233; *p* = 0.036). There was a statistically significant negative correlation between IL-8 and albumin (moderate correlation; rho = −0.498; *p* < 0.001) and time to auto-HSCT (weak correlation; rho = −0.294; *p* = 0.009).

None of the studied demographic and clinical variables correlated significantly with the VEGF concentration or the MCP-1 concentration.

Table 4 includes detailed data on the correlations between the selected demographic, clinical, and laboratory variables and the tested cytokines.

### 3.6. Survival Analysis

#### 3.6.1. Progression-Free Survival

**Figure 3:**
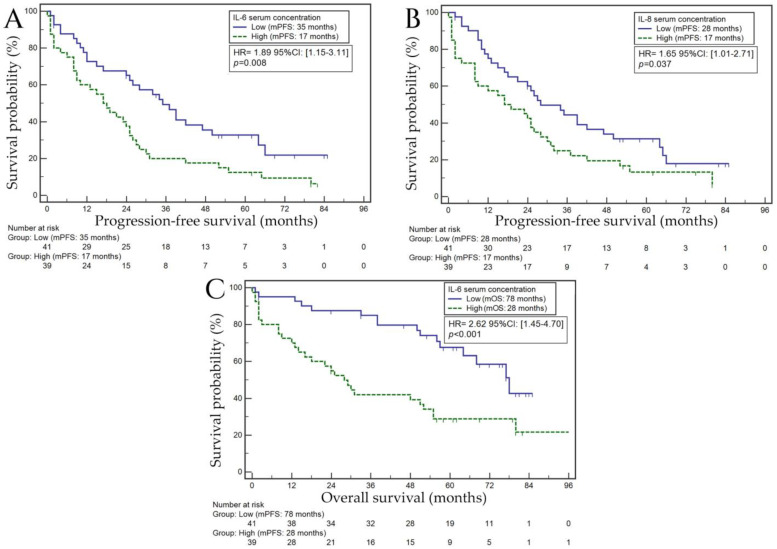
Kaplan–Meier curves representing the probability of progression-free survival depending on the concentration of IL-6 (**A**), progression-free survival depending on the concentration of IL-8 (**B**), and overall survival depending on the concentration of IL-6 (**C**) in the blood serum of patients from the study group. Abbreviations: mPFS—median progression-free survival; mOS—median overall survival.

Variables that are established as negative prognostic factors in MM, such as higher ISS stage (2 or 3 vs. 1; median PFS: 17 vs. 52 months; HR = 2.92; *p* < 0.001), worse renal function (serum creatinine ≥ 2.0 mg/dL, vs. <2.0 mg/dL; median PFS: 15 vs. 28 months; HR = 2.12; *p* = 0.008), and 17p deletion (present vs. no deletion; median PFS: 11 vs. 37 months; HR = 2.72; *p* = 0.004), were indeed correlated with a shorter PFS. In individuals who did not undergo auto-HSCT, the risk of PFS shortening was over 2.5-fold higher than in those who underwent this procedure (median PFS: 39 vs. 13 months; HR = 2.61; *p* < 0.001). Subjects with a high concentration of IL-6 had a nearly two-fold higher risk of PFS reduction than those with a low concentration of this cytokine (median PFS: 17 vs. 35 months; HR = 1.89; *p* = 0.008; Figure 3A). The risk of PFS reduction was over 1.5 times higher in patients with a high IL-8 concentration compared with patients with a low concentration of this cytokine (median PFS: 17 vs. 28 months; HR = 1.65; *p* = 0.037; Figure 3B). In our multivariable analysis, the following variables demonstrated significance associated with a higher risk of PFS shortening: A/B renal function (HR = 2.29; *p* = 0.051) and 17p deletion (HR = 2.44; *p* = 0.042). The female sex was significantly independently related to a lower risk of PFS shortening (HR = 0.99; *p* = 0.002). Supplementary Table S1 provides detailed data on the relationship between the selected demographic and clinical variables and PFS in the study group.

#### 3.6.2. Overall Survival

Men presented with a more than two-fold higher risk of OS shortening than women (median OS: 38 vs. 78 months; HR = 2.17; *p* = 0.007). Well-known negative prognostic factors in our study also resulted in an OS shortening, such as higher ISS stage (2 or 3 vs. 1; median OS: 48 months vs. not reached; HR = 2.99; *p* = 0.004), worse renal function (B vs. A; median OS: 18 vs. 68 months; HR = 2.78; *p* = 0.001), and 17 p deletion (present vs. no deletion, median OS: 28 vs. 80 months; HR = 2.83; *p* = 0.018). In patients who did not undergo auto-HSCT, a nearly three-fold higher risk of OS shortening was observed compared with those who underwent this procedure (median OS: 30 months vs. not achieved; HR = 2.79; *p* < 0.001). Patients who lost weight before treatment showed an approximately two-fold higher risk of OS shortening than those who did not experience changes in body mass (median OS: 33 vs. 78; HR = 2.11; *p* = 0.009). We showed that the risk of OS shortening in individuals without hypertension was 51% lower than in those with this disease (median OS: 78 vs. 51 months; HR = 0.49; *p* = 0.023). The risk of OS shortening was over 2.5 times higher in patients with a high concentration of IL-6 compared with those with a low concentration of this cytokine (median OS: 28 vs. 78 months; HR = 2.62; *p* < 0.001; Figure 3C). Our multivariate analysis confirmed that male gender (HR = 2.46; *p* = 0.01), higher disease stage (ISS 2 or 3) (HR = 3.14; *p* = 0.005), the occurrence of 17p deletion (HR = 2.80; *p* = 0.048), no auto-HSCT (HR = 1.90; *p* = 0.013), and a high concentration of IL-6 (HR = 2.79; *p* = 0.039) were independent unfavorable prognostic factors. Supplementary Table S1 includes detailed data on the relationship between the selected demographic and clinical factors and OS in the study group.

### 3.7. The Usefulness of the Tested Cytokines in Differentiating Various Clinical Conditions

Supplementary Table S2 includes detailed data on the usefulness of the tested cytokines in differentiating between various clinical conditions.

The concentration of IL-6 demonstrated 50% sensitivity and 81% specificity in differentiating disease stages according to the ISS classification (AUC = 0.64; *p* = 0.012). In addition, the concentration of IL-6 presented 67% sensitivity and 77% specificity in predicting the occurrence of weight loss before treatment (AUC = 0.70; *p* < 0.001).

The concentration of IL-8 demonstrated 55% sensitivity and 72% specificity in differentiating disease stages according to the ISS classification (AUC = 0.66; *p* = 0.021); 80% sensitivity and 77% specificity in differentiating the grades of renal disease (AUC = 0.84; *p* < 0.001); 75% sensitivity and 85% specificity in differentiating ECOG performance status (AUC = 0.75; *p* = 0.010); 65% sensitivity and 80% specificity in distinguishing patients who receive supportive care (AUC = 0.77; *p* < 0.001); 76% sensitivity and 80% specificity in differentiating the severity of anemia before treatment (AUC = 0.79; *p* < 0.001); and 64% sensitivity and 66% specificity in predicting the feasibility of auto-HSCT (AUC = 0.68; *p* = 0.003).

The VEGF concentration demonstrated 93% sensitivity and 59% specificity in differentiating the grades of renal disease (AUC = 0.73; *p* < 0.001).

We found no statistically significant utility of MCP-1 in differentiating the various clinical conditions.

## 4. Discussion

A sophisticated cytokine network actively participates in various malignancies, including MM. Furthermore, cytokines such as interleukins, growth factors, tumor necrosis factor (TNF)-alpha, interferon (IFN)-gamma, and MCP-1 are known to regulate biological processes and are associated with disease progression [37,38]. We investigated whether demographic and clinical factors in our study group affected the concentrations of the tested cytokines and whether these dependencies were statistically significant. In short, we sought to determine the source of the indirect relationship between demographic and clinical factors and treatment outcomes. We acquired important data on the cases in which the relationship between the tested cytokines and treatment results could depend on the abovementioned factors. In our study, the measurements of selected concentrations of chemokines and pro-inflammatory cytokines was performed at the time of MM diagnosis, before the start of treatment. Therefore, we did not consider the impact of individual chemotherapy regimens on the levels of the studied cytokines. Our study demonstrated a relationship between serum IL-6, IL-8, and VEGF and clinical features such as treatment response and survival in patients newly diagnosed with MM.

IL-6 has a crucial role in the progression and assessment of prognosis in MM patients [39,40]. Gernone et al. carried out a study confirming that high IL-6 levels can be a factor in differentiating MM from a monoclonal gammopathy of undetermined significance (MGUS) [41]. Furthermore, a high cellular expression of IL-6 mRNA in MGUS patients may predict the development of MM [42]. Moreover, IL-6 has been reported to stimulate the in vitro growth of new myeloma cells isolated from patients [39]. IL-6 is a factor that determines the survival of MM cells through the inhibition of apoptosis [43]. Secretion of IL-6 by bone marrow stromal cells (BMSCs) enhances transcription of the anti-apoptotic factor MCL-1 [44]. Moreover, there are reports that IL-6 plays a key role in the development of drug resistance, including through Dickkopf-1 (DKK1) regulatory mechanisms [45].

The effectiveness of drugs inhibiting IL-6 activity in MM therapy was analyzed. Previous research confirmed that anti–IL–6 antibodies had anti-tumor effects in MM [46]. Other analyses showed consistent results, with elevated IL-6 and sIL-6R reflecting disease activity and indicating a poor prognosis. A positive response to treatment was correlated with lower levels of IL-6 in patients with MM, whereas advanced disease corresponded to significantly elevated levels of the cytokine [47]. From a clinical standpoint, researchers have suggested a potential role of IL-6 in mediating renal failure through the tumor cell burden and suppressing osteoblastic activity [42,48,49,50].

In our study, we found that patients from the countryside had a significantly higher concentration of IL-6 than those coming from the city (*p* = 0.047). These results stand in unison with previous findings, where the authors suggested that IL-6 circulation increases considerably in response to physical activity depending on its duration and intensity, as well as the endurance capacity of the individuals [51,52,53]. It is likely that the more active and physical lifestyles of people living in the countryside predispose them to increased levels of IL-6 [54]. We demonstrated that in former smokers, the concentration of IL-6 was significantly higher than in those who never smoked (*p* = 0.032). This confirms the previous reports stating that smoking results in a strong and durable inflammatory response, including an effect on IL-6 [55].

In our study, we observed significantly higher concentrations of IL-6 in patients in ISS stage 2 or 3 than in stage 1. Our observations are consistent with previous reports. Chakraborty B. et al. observed a positive correlation between the ISS stage and serum IL-6 concentration (r^2^  =  0.4, *p*  =  0.01) [56].

We further observed that the patients who lost weight before their MM diagnosis also presented with significantly higher concentrations of the cytokine than those whose body weight remained unchanged. These findings are in line with previous reports showing that the circulating level of IL-6 correlates with weight loss in cancer patients, as this cytokine can act alone as a driver of systemic inflammation in cancer cachexia, inducing anorexia, body mass loss, and an acute-phase protein response [57,58,59,60,61].

Interestingly, our study reported that patients with previous exposure to chemical or physical agents had significantly higher concentrations of IL-6 (*p* = 0.038). Exposure to chemicals was proven to induce chronic inflammation by triggering the activation of IL-6 amplifier and its production [62,62]. Although the etiology of MM remains unclear, the importance of environmental factors, type of work, lifestyle, and chronic exposure to pesticides and chemicals is emphasized in the development of the disease. Jiayan Gu et al., in their retrospective analysis of cytokine levels in patients newly diagnosed with MM, reported that the complete response (CR) rate in subjects with lower serum IL-6 levels was significantly higher (48.28% vs. 27.63%) than those with higher levels. Moreover, the overall response rate (ORR) in people with serum IL-6 below the median was significantly higher than in those whose IL-6 level was above the median (86.21% vs. 59.21%) [11]. In our study, we observed significantly lower concentrations of IL-6 in patients with an early response to treatment (CR, VGPR, PR, or MR) compared to those who did not respond after two cycles of CTH (PD or SD). In addition, we found that individuals with a significantly higher level of IL-6 had a nearly two-fold higher risk of PFS reduction than those with a low level of this cytokine. Moreover, patients with IL-6 levels above the median presented with a more than 2.5-fold higher risk of OS shortening than those with low levels of this cytokine (median OS: 28 vs. 78 months; HR = 2.62). The multivariate analysis confirmed that a high level of IL-6 is an independent unfavorable prognostic factor (HR = 2.79).

IL-6 involvement is observed in the early stages of the disease, where the myeloma interacts with the stroma and it plays a crucial role in cancer cell survival; thus, hypothetically, anti-IL-6 therapy in the early stages of the disease may have promising results [46]. Siltuximab is a monoclonal anti-IL-6 antibody (mAb) that has been studied in several clinical trials [63,64]. In a trial of 286 patients with relapsed refractory MM, adding siltuximab to bortezomib therapy did not improve the PFS or OS. The authors reported that this combination treatment led to a shorter time to response (TTR) and a longer duration of response (DoR) than bortezomib alone [64]. In addition, a clinical trial (NCT01309412) sought to determine whether siltuximab could improve symptoms after autologous stem cell transplant in patients with MM and systemic AL amyloidosis. The study was terminated due to safety concerns. Tocilizumab is another humanized anti-IL-6R mAb. However, a clinical trial (NCT02447055) for treating myeloma using tocilizumab after allogeneic stem cell transplantation was discontinued due to a low accrual rate [65]. To date, randomized clinical trials have shown no efficacy of IL-6-targeted therapies in MM. Nevertheless, IL-6 seems to be an important prognostic marker for patients with MM.

Shapiro et al. suggested that the expression of endogenous CD28 in myeloma cells increases IL-8 production in patients with MM, consequently promoting myeloma metastasis [66]. Pellegrino et al. published a study showing that IL-8 may induce the proliferation and chemotaxis of MM cell lines and patient plasma cells [67]. IL-8 is involved in the disturbance of bone homeostasis in various types of cancer. Hence, it has been suggested that anti-IL-8 therapies could be helpful in the prevention of MM-induced osteolysis [10].

In our study, the level of IL-8 was significantly higher in patients over 65, those in ISS stage 2 or 3, and those with kidney impairment and anemia. According to the literature, IL-8 levels do not increase in elderly patients in the general population [68]. However, there were also reports of elderly patients overproducing IL-8 upon stimulation [69]. We were not able to find any reliable reports of IL-8 levels in myeloma-related kidney injury. One could argue that the increased level of IL-8 in renal failure might be the result of its lower clearance via the kidneys. However, in the reported studies, the increased level of IL-8 in kidney diseases was attributed to acute or chronic inflammation rather than the slowed disposal of that cytokine from circulation through the urinary system [70,71,72,73]. Notably, the ISS stage can be influenced by kidney function, as the beta-2-microglobulin (which is one of the two markers analyzed in the ISS scale) level is dependent on the glomerular filtration rate [74,75,76,77,78]. Although no research has been published that assessed the impact of pretreatment IL-8 levels and pretreatment anemia, Tripathy et al. published a study showing that the bone marrow and peripheral blood of patients with aplastic anemia had increased levels of IL-8, and this cytokine correlated with disease severity [79]. In fact, although many researchers have confirmed an increase in IL-8 concentrations in patients with MM, it has not yet been proven that IL-8 is significantly associated with PFS and OS [80].

We showed that the serum IL-8 concentration could be a factor predictive of a response to chemotherapy. However, the obtained results should be treated with caution due to the relatively small study group.

Ribas et al. and Palta et al. suggested that increased production of VEGF and, thus, increased angiogenesis may be associated with more aggressive disease [81,82]. Observations by Raimondo et al. showed that levels of VEGF in the bone marrow were markedly higher than in the peripheral blood of patients with MM. Moreover, its levels were lower in stage 1 of the disease than in stages 2 and 3. Furthermore, VEGF levels were correlated with features of disease activity, such as C-reactive protein and β2-microglobulin [83]. Saltarella I et al. confirmed that a high VEGF concentration in myeloma patients is associated with an unfavorable prognosis and shorter PFS and OS [80].

In our study, patients with “B” kidney function had significantly higher VEGF concentrations than patients with “A” renal function. Furthermore, patients who responded to CTH had significantly lower VEGF concentrations. Furthermore, the VEGF concentration had 93% sensitivity and 59% specificity in differentiating the grades of renal disease in our study.

Notably, novel agents against MM have significantly improved the response to therapy and survival rates. Drugs such as proteasome inhibitors or immunomodulators have exhibited marked antiangiogenic effects. Pazopanib is a multi-kinase inhibitor with activity against VEGFR and other receptors [84]. Nevertheless, three clinical trials failed to produce meaningful clinical responses with single-agent regimens of VEGF inhibitors in MM [85,86,87].

No studies have yet explored associations between plasma MCP-1 levels, angiogenesis, and the main clinical features in newly diagnosed, untreated myeloma patients, such as anemia, renal dysfunction, and bone disease, which was the aim of the present pilot study. Valković et al. demonstrated a significant association between plasma MCP-1 levels and the main clinical features of MM. They showed that patients with higher chemokine levels exhibited more severe bone disease, renal impairment, and anemia. The authors warn, however, that an association between increased MCP-1 plasma levels and creatinine concentration may result from the decreased renal clearance of this chemokine in patients with renal failure and thus should be interpreted with caution [88]. Liu et al. showed that MM cells increase the production of MCP-1 by bone marrow stromal cells, which then enhances osteoclast formation [89]. Salcedo et al. demonstrated that MCP-1 might induce blood vessel formation in vivo, which can be inhibited by using neutralizing antibodies against MCP-1 [24]. Our study did not find any statistical significance of MCP-1 in patients with MM. However, an analysis of bone marrow aspirates may result in more significant outcomes.

Our study demonstrated associations between the analyzed factors and patients’ OS. Multivariate analysis revealed several independent unfavorable prognostic factors: male gender (HR = 2.46; *p* = 0.0104), higher disease stage (ISS 2 or 3) (HR = 3.14; *p* = 0.006), the presence of a 17p deletion (HR = 2.80; *p* = 0.049), no auto-HSCT (HR = 1.90; *p* = 0.013), and a high concentration of IL-6 (HR = 2.79; *p* = 0.0397). We observed that male sex was associated with a significantly higher risk of OS shortening (median OS: 38 vs. 78 months; HR = 2.17; *p* = 0.008). These results differ from previous observations. Based on the National Cancer Institute’s Surveillance, Epidemiology, and End Results (SEER) Program and the MRC Myeloma IX trial, gender is not associated with any differences in PFS or OS [90,91]. We found that the disease stage, according to ISS classification, was an independent prognostic factor for OS. The risk of its reduction was over three times higher in subjects with stage 2 and 3 disease than those with stage 1. The median survival based on the ISS in our study was stage 1: 62 months, stage 2: 44 months, and stage 3: 29 months. In our study, subjects with del (17p) were almost three times more likely to experience an OS reduction. We demonstrated improved OS with auto-HSCT incorporation into standard therapy. Our observations are consistent with those previously reported and further validate our cohort [28,74,92,93].

Interestingly, among the analyzed proteins, namely, IL-6, IL-8, VEGF, and MCP-1, we confirmed that IL-6 is an independent prognostic factor for patients with MM.

This study has some limitations. Firstly, the study group was relatively small and was only representative of the Polish community. Therefore, the results might not correspond to all patient populations. Nonetheless, based on the median age of our patients, the male to female ratio, and the type of diagnosed disease, our group was very similar to worldwide statistics for MM [94]. Secondly, we did not perform a functional analysis that would define the importance of the studied cytokines in the pathogenesis of the disease. Thirdly, only baseline cytokines levels were obtained, whereas serial measurements through the therapy course could provide valuable data. Moreover, more than one combination of treatments was used as a standard of care during the material collection process. During statistical analysis, we also used multiple comparisons. Finally, comparisons with data in the literature are limited due to a lack of similar research.

Nonetheless, despite these limitations, our study presents meaningful results. From a clinical point of view, assessing predictive and prognostic factors at diagnosis could improve the development of individualized therapies. Based on the results of this study, further studies can be designed to evaluate the concentrations of cytokines in the blood and supernatant of the bone marrow before and during the treatment.

Daily clinical practice shows numerous patients whose disease course is very different from the course predicted by widely used scales and classifications. With multiple new drugs available, selecting the first-line treatment in patients with MM requires careful analysis of all known predictive and prognostic factors.

Our research offers insight into the intricate cytokine network in MM and its potential role in disease progression and treatment outcomes. It emphasizes the need for individualized treatment approaches, considering the patient’s cytokine profile. Understanding these cytokine profiles may help in the early detection of MM, risk stratification, and prognosis, potentially leading to more tailored and effective treatment strategies. This study’s findings could pave the way for future research and therapeutic interventions, improving the clinical management of MM patients.

Understanding the profiles of the cytokines in the MM microenvironment can provide valuable information. They can serve as biomarkers that are helpful in diagnosing MM and its further risk stratification. In addition, proper utilization of the knowledge of pretreatment levels of analyzed cytokines could provide additional information about the clinical course of the disease.

## 5. Conclusions

Based on this study’s results, high levels of IL-6, IL-8, and VEGF have significant predictive value for certain clinical conditions or outcomes among newly diagnosed MM patients. We found that a high concentration of IL-6 was associated with more advanced disease, increased weight loss, and shorter median PFS and OS. Patients with elevated IL-8 levels were at more advanced stages of the disease, exhibited poorer kidney function, and faced a higher risk of PFS reduction. Moreover, high VEGF concentrations were found in subjects with worse kidney function. Patients with an early response to chemotherapy had significantly lower concentrations of IL-6, IL-8, and VEGF. Furthermore, a high IL-6 level emerged as a significant and independent negative prognostic marker. These findings pave the way for future research, but they should be validated in a more extensive patient cohort.

## Figures and Tables

**Table 1:** Characteristics of the study group.

Variable	Study Group (*n* = 82)
**Sex** Women Men	40 (48.8%) 42 (51.2%)
**Age [years]** **<65** **>65**	40 (48.8%) 42 (51.2%)
**Smoking** No Yes Ex-smoker	59 (72.8%) 9 (11.1%) 13 (16.0%)
**Diagnosis** Disease with monoclonal protein present Light chain disease	72 (87.8%) 10 (12.2%)
**A type of monoclonal protein** IgA IgG	20 (27.8%) 52 (72.2%)
**Light chains** Kappa Lambda	48 (58.5%) 34 (41.5%)
**ISS Stage** 1 2 3	23 (28.4%) 23 (28.4%) 35 (43.2%)
A/B renal function * A B	67 (81.7%) 15 (18.3%)
**ECOG scale** 0 1 2 3 4	8 (9.8%) 34 (41.5%) 25 (30.5%) 12 (14.6%) 3 (3.7%)
**Cytogenetic abnormalities** **Presence of abnormality/no. of evaluated patients** **17p deletion**	9/54
**Translocation (4; 14)**	6/54
**Translocation (14; 16)**	3/54
**A type of CTH** CTD V(C)D, VMP, PAD VTD Cyclophosphamide	38 (46.3%) 29 (35.4%) 14 (17.1%) 1 (1.2%)
**auto-HSCT** No Yes	45 (59.2%) 31 (40.8%)

Abbreviations: Auto-HSCT—Autologous hematopoietic stem cell transplantation; C—Cyclophosphamide; CTH—Chemotherapy; D—Dexamethasone; ECOG—Eastern Cooperative Oncology Group; ISS—International Staging System; P—Prednisolone; PAD—Bortezomib, adriamycin, dexamethasone; T—Thalidomide; V—Bortezomib, WHO—World Health Organization. * Renal function A—creatinine level < 2.0 mg/dL; Renal function B—creatinine level ≥ 2.0 mg/dL.

**Table 2:** Comparison of the concentrations of the assessed cytokines depending on the demographic and clinical variables.

Variable	Study Group (*n* = 82)	IL-6 [fg/mL]		IL-8 [fg/mL]		VEGF [pg/mL]		MCP-1 [pg/mL]	
		Median [Interquartile Range]	*p*	Median [Interquartile Range]	*p*	Median [Interquartile Range]	*p*	Median [Interquartile Range]	*p*
**Sex** Women Men	40 (48.8%) 42 (51.2%)	0.10 [0.10–1423.36] 1241.92 [0.10–7534.10]	0.187	17,285.32 [10,817.30–28,443.36] 12,519.28 [5973.07–24,851.20]	0.1576	34.23 [24.06–57.54] 44.02 [26.30–74.28]	0.372	163.65 [109.59–267.43] 159.37 [101.74–216.58]	0.301
**Age > 65** Below the median Above the median	40 (48.8%) 42 (51.2%)	0.10 [0.10–9005.17] 631.53 [0.10–16,546.52]	0.519	10,768.45 [6671.11–16,091] 18,836.52 [11,748.83–42,411.06]	0.005 *	39.73 [26.93–60.07] 34.23 [23.85–62.61]	0.698	159.36 [101.54–221.77] 163.65 [108.07–250.70]	0.390
**Place of residence** City Countryside	49 (59.8%) 33 (40.2%)	0.10 [0.10–1611.02] 756.68 [0.10–18,932.64]	0.047 *	14,380.48 [1692.04–23,876] 18,364.81 [6961.40–34,823.52]	0.504	36.46 [24.13–63.22] 40.69 [25.41–55.57]	0.935	169.30 [104.75–233.36] 147.59 [108.03–244.83]	0.942
**Exposure to chemical or physical agents** No Yes	54 (68.4%) 25 (31.6%)	0.10 [0.10–8807.95] 2389.75 [0.10–19,950.49]	0.038 *	13,534.43 [7056.82–24,843.10] 18,364.81 [6961.40–29,343.91]	0.363	35.08 [25.07–59.27] 47.74 [25.12–55.35]	0.847	157.07 [102.25–228.62] 161.66 [107.35–246.56]	0.661
**Smoking** No Yes	59 (72.8%) 22 (27.2%)	0.10 [0.10–8310.60] 3873.71 [0.10–15,071.58]	0.032 *	13,899.46 [7229.50–24,851.20] 17,681.67 [6870.38–33,111.58]	0.383	40.23 [22.81–58.72] 36.67 [29.89–72.05]	0.379	172.48 [108.41–224.37] 147.67 [102.43–212.71]	0.401
**Diagnosis** Secretory disease Light chain disease	72 (87.8%) 10 (12.2%)	0.10 [0.10–10,114.65] 4197.73 [0.10–15,514.61]	0.525	14,496.47 [7051.18–24,653.44] 31,684.17 [11,029.50–49,241.79]	0.214	35.51 [24.48–59.08] 48.28 [30.43–65.51]	0.405	165.12 [113.65–237.98] 104.13 [83.04–202.21]	0.115
**A type of monoclonal protein** IgA IgG	20 (27.4%) 53 (72.6%)	0.10 [0.10–9452.95] 296.51 [0.10–10,276.49]	0.715	15,641.41 [11,472.77–22,428.66] 14,380.48 [6719.68–27,525.48]	0.542	31.47 [23.07–50.13] 39.94 [26.05–66.49]	0.274	153.71 [109.85–235.67] 172.48 [112.09–236.73]	0.646
**Light chains** Kappa Lambda	48 (58.5%) 34 (41.5%)	0.10 [0.10–10,114.65] 1819.51 [0.10–18,603.44]	0.161	14,264.49 [6674.67–22,816.84] 17,825.06 [9555.49–33,111.58]	0.286	35.08 [23.58–60.50] 44.21 [29.89–58.72]	0.389	150.32 [108.38–234.08] 173.16 [101.35–248.97]	0.822
**ISS Stage** 1 2 or 3	23 (28.4%) 58 (71.6%)	0.10 [0.10–1140.65] 1049.94 [0.10–15,860.88]	0.045 *	10,976.64 [5758.83–18,760.78] 17,219.26 [10,350.34–31,515.43]	0.032 *	32.70 [22.70–48.39] 47.54 [25.80–74.28]	0.133	154.50 [97.04–227.33] 162.65 [108.41–243.46]	0.324
**A/B renal function** A B	67 (81.7%) 15 (18.3%)	0.10 [0.10–8310.60] 8860.32 [0.10–23,928.07]	0.177	12,186.41 [6538.78–19,816.70] 46,279.57 [21,276.26–62,116.15]	<0.001 *	32.83 [23.45–56.79] 50.15 [48.01–96.14]	0.004 *	153.71 [107.08–232.49] 212.71 [107.05–310.70]	0.261
**Weight loss** No Yes	41 (50.6%) 40 (49.4%)	0.10 [0.10–694.10] 4076.92 [0.10–19,980.73]	<0.001 *	11,989.80 [6931.06–21,387.32] 16,761.99 [9766.08–32,729.32]	0.166	44.75 [27.31–74.31] 33.49 [23.13–53.02]	0.157	168.62 [107.95–236.44] 151.98 [101.54–230.60]	0.507
**17p deletion** No Yes	45 (83.3%) 9 (16.7%)	0.10 [0.10–4828.74] 8310.60 [473.67–21,877.49]	0.025 *	14,264.49 [6208.55–24,257.92] 15,736.18 [7064.37–22,405.28]	0.745	38.77 [25.41–59.64] 37.84 [24.11–48.80]	0.458	165.12 [106.06–232.78] 107.08 [84.86–142.97]	0.060
**Translocation t (4; 14)** No Yes	48 (88.9%) 6 (11.1%)	0.10 [0.10–7387.02] 5228.21 [0.10–19,920.26]	0.268	14,380.48 [6553.88–23,230.32] 15,006.21 [10,793.25–24,060.16]	0.731	39.27 [29.28–57.02] 20.79 [14.85–47.74]	0.191	152.33 [101.54–221.26] 179.80 [106.91–316.11]	0.449
**Translocation t (14; 16)** No Yes	51 (94.4%) 3 (5.6%)	756.68 [0.10–10,114.65] 0.10 [–]	0.256	15,546.65 [6674.67–24,653.44] 9555.49 [–]	0.345	38.77 [24.65–58.11] 32.91 [–]	0.623	147.59 [101.44–223.60] 179.58 [–]	0.473
**Degree of anemia before treatment (WHO)** No Yes	21 (25.6%) 61 (74.4%)	0.10 [0.10–1581.35] 830.03 [0.10–17,232.16]	0.053	7229.50 [5919.51–10,852.31] 18,798.65 [11,736.45–32,729.32]	<0.001 *	32.91 [19.83–53.73] 40.23 [25.32–69.89]	0.142	150.36 [100.27–196.99] 164.38 [107.29–247.55]	0.290

* Statistically significant result. Abbreviations: IL-6—interleukin-6; IL-8—interleukin-8; MCP-1—angiogenic chemokine monocyte chemoattractant protein-1; VEGF—vascular endothelial growth factor.

**Table 3:** Comparison of the baseline concentrations of the tested cytokines depending on the response to CTH.

Variable	Study Group (*n* = 82)	IL-6 [fg/mL]		IL-8 [fg/mL]		VEGF [pg/mL]		MCP-1 [pg/mL]	
		Median [Interquartile Range]	*p*	Median [Interquartile Range]	*p*	Median [Interquartile Range]	*p*	Median [Interquartile Range]	*p*
**Response to CTH after two cycles** No Yes	14 (18.7%) 61 (81.3%)	17,777.91 [1165.03–38,143.62] 0.10 [0.10–4030.78]	<0.001 *	36,113.32 [18,364.81–51,165.99] 12,096.70 [6719.68–18,897.67]	<0.001 *	53.97 [31.81–103.41] 34.65 [23.40–54.54]	0.040 *	148.88 [117.66–328.97] 159.37 [101.54–233.94]	0.525
**Response to CTH during the treatment period** No Yes	10 (13.0%) 65 (87.0%)	1147.96 [0.10–10,252.95] 0.10 [0.10–8448.03]	0.728	19,277.90 [10,350.34–24,060.16] 12,397.80 [6795.03–22,419.88]	0.374	44.34 [31.81–117.28] 37.84 [24.73–59.64]	0.233	153.85 [120.95–212.71] 157.09 [101.64–240.51]	0.827

* Statistically significant result.

**Table 4:** Correlations between the selected demographic, clinical, and laboratory variables and the tested cytokines.

Variable	*n*	IL-6 [fg/mL]		IL-8 [fg/mL]		VEGF [pg/mL]		MCP-1 [pg/mL]	
		rho	*p*	rho	*p*	rho	*p*	rho	*p*
**Demographic and clinical**									
**Age at diagnosis**	81	0.095	0.398	0.297	0.007 *	−0.036	0.748	0.006	0.955
**ISS Stage**	80	0.245	0.028	0.242	0.030 *	0.170	0.132	0.112	0.325
**Percentage of plasmacytes in the bone marrow**	76	0.059	0.611	0.101	0.383	0.060	0.605	0.043	0.715
**The number of lytic bone lesions**	80	0.121	0.286	−0.094	0.405	−0.136	0.231	−0.165	0.144
**eGFR before treatment (mL/min/1.73 m^2^)**	81	−0.246	0.026	−0.397	<0.001 *	−0.155	0.166	0.013	0.906
**The grade of kidney disease**	81	0.226	0.042	0.396	<0.001 *	0.122	0.279	−0.003	0.981
**A/B renal function**	81	0.217	0.051	0.466	<0.001 *	0.319	0.003 *	0.126	0.261
**ECOG scale**	81	0.369	<0.001 *	0.445	<0.001 *	0.082	0.466	0.029	0.799
**BMI**	54	−0.086	0.537	−0.113	0.414	0.060	0.664	0.152	0.272
**Weight loss**	80	0.410	<0.001 *	0.145	0.199	−0.204	0.069	−0.095	0.399
**Grade of anemia before treatment according to WHO**	81	0.360	0.001 *	0.520	<0.001 *	0.247	0.026 *	0.074	0.512
**Laboratory**									
**HGB (g/dL)**	81	−0.467	<0.001 *	−0.554	<0.001 *	−0.180	0.108	−0.075	0.503
**LYM before treatment (K/uL)**	81	0.049	0.661	−0.120	0.286	0.110	0.330	0.031	0.783
**NEU before treatment (K/uL)**	81	0.178	0.112	0.045	0.689	0.070	0.533	−0.104	0.356
**NEU/LYM**	81	0.090	0.426	0.112	0.320	−0.003	0.979	−0.106	0.348
**PLT (K/uL)**	81	−0.003	0.978	−0.118	0.294	0.091	0.421	−0.014	0.902
**MPV**	81	0.037	0.744	0.150	0.183	−0.097	0.387	−0.102	0.364
**Albumin**	81	−0.466	<0.001 *	−0.498	<0.001 *	−0.132	0.239	0.046	0.684
**Creatinine (mg/dL)**	81	0.196	0.079	0.321	0.004 *	0.229	0.040 *	0.071	0.531
**β2-microglobulin (ng/L)**	79	0.301	0.007	0.415	0.001 *	0.168	0.138	0.152	0.180
**LDH (IU/L)**	70	−0.136	0.260	−0.090	0.459	0.081	0.507	−0.172	0.155
**Calcium (mmol/L)**	81	0.074	0.511	−0.181	0.106	−0.073	0.515	−0.093	0.406
**CRP (mg/L)**	77	0.503	<0.001 *	0.361	0.002 *	0.258	0.023 *	−0.021	0.855
**Cytokines**									
**IL-8**	81	0.580	<0.001 *	X	X	X	X	X	X
**IL-6**	81	X	X	X	X	X	X	X	X
**VEGF**	81	0.112	0.320	0.233	0.036 *	X	X	X	X
**MCP-1**	81	−0.023	0.840	0.210	0.060	−0.016	0.888	X	X
**Related to treatment**									
**Time to auto-HSCT (months)**	78	−0.196	0.086	−0.294	0.009 *	−0.073	0.527	−0.138	0.229

* Statistically significant result.

## Data Availability

Data are contained within the article.

## Supplementary Materials

The following supporting information can be downloaded at: https://www.mdpi.com/article/10.3390/biomedicines11113012/s1.

## References

[1] Van de Donk N.W.C.J., Pawlyn C., Yong K.L. (2021). Multiple Myeloma. Lancet.

[2] Seidl S., Kaufmann H., Drach J. (2003). New Insights into the Pathophysiology of Multiple Myeloma. Lancet Oncol..

[3] Jasrotia S., Gupta R., Sharma A., Halder A., Kumar L. (2020). Cytokine Profile in Multiple Myeloma. Cytokine.

[4] Musolino C., Allegra A., Innao V., Allegra A.G., Pioggia G., Gangemi S. (2017). Inflammatory and Anti-Inflammatory Equilibrium, Proliferative and Antiproliferative Balance: The Role of Cytokines in Multiple Myeloma. Mediat. Inflamm..

[5] Yang W.C., Lin S.F. (2015). Mechanisms of Drug Resistance in Relapse and Refractory Multiple Myeloma. Biomed. Res. Int..

[6] Papadas A., Asimakopoulos F. (2018). Mechanisms of Resistance in Multiple Myeloma. Handb. Exp. Pharmacol..

[7] Dewald J.H., Colomb F., Bobowski-Gerard M., Groux-Degroote S., Delannoy P. (2016). Role of Cytokine-Induced Glycosylation Changes in Regulating Cell Interactions and Cell Signaling in Inflammatory Diseases and Cancer. Cells.

[8] Coussens L.M., Werb Z. (2002). Inflammation and Cancer. Nature.

[9] Möller C., Strömberg T., Juremalm M., Nilsson K., Nilsson G. (2003). Expression and Function of Chemokine Receptors in Human Multiple Myeloma. Leukemia.

[10] Aggarwal R., Ghobrial I.M., Roodman G.D. (2006). Chemokines in Multiple Myeloma. Exp. Hematol..

[11] Gu J., Huang X., Zhang Y., Bao C., Zhou Z., Jin J. (2021). Cytokine Profiles in Patients with Newly Diagnosed Multiple Myeloma: Survival Is Associated with IL-6 and IL-17A Levels. Cytokine.

[12] Ogiya D., Liu J., Ohguchi H., Kurata K., Samur M.K., Tai Y.T., Adamia S., Ando K., Hideshima T., Anderson K.C. (2020). The JAK-STAT Pathway Regulates CD38 on Myeloma Cells in the Bone Marrow Microenvironment: Therapeutic Implications. Blood.

[13] Waugh D.J.J., Wilson C. (2008). The Interleukin-8 Pathway in Cancer. Clin. Cancer Res..

[14] Zeilhofer H.U., Schorr W. (2000). Role of Interleukin-8 in Neutrophil Signaling. Curr. Opin. Hematol..

[15] Shahzad A., Knapp M., Lang I., Köhler G. (2010). Interleukin 8 (IL-8)-a Universal Biomarker?. Int. Arch. Med..

[16] Yuan A., Chen J.J.W., Yao P.L., Yang P.C. (2005). The Role of Interleukin-8 in Cancer Cells and Microenvironment Interaction. Front. Biosci..

[17] Carmeliet P. (2005). Angiogenesis in Life, Disease and Medicine. Nature.

[18] Podar K., Anderson K.C. (2005). The Pathophysiologic Role of VEGF in Hematologic Malignancies: Therapeutic Implications. Blood.

[19] Vacca A., Ria R., Ribatti D., Semeraro F., Djonov V., Di Raimondo F., Dammacco F. (2003). A Paracrine Loop in the Vascular Ebnothelial Growth Factor Pathway Triggers Tumor Angiogenesis and Growth in Multiple Myeloma. Haematologica.

[20] Podar K., Tai Y.T., Davies F.E., Lentzsch S., Sattler M., Hideshima T., Lin B.K., Gupta D., Shima Y., Chauhan D. (2001). Vascular Endothelial Growth Factor Triggers Signaling Cascades Mediating Multiple Myeloma Cell Growth and Migration. Blood.

[21] Ria R., Reale A., De Luisi A., Ferrucci A., Moschetta M., Vacca A. (2011). Bone Marrow Angiogenesis and Progression in Multiple Myeloma. Am. J. Blood Res..

[22] Baggiolini M., Loetscher P. (2000). Chemokines in Inflammation and Immunity. Immunol. Today.

[23] Vande Broek I., Asosingh K., Vanderkerken K., Straetmans N., Van Camp B., Van Riet I. (2003). Chemokine Receptor CCR2 Is Expressed by Human Multiple Myeloma Cells and Mediates Migration to Bone Marrow Stromal Cell-Produced Monocyte Chemotactic Proteins MCP-1, -2 and -3. Br. J. Cancer.

[24] Salcedo R., Ponce M.L., Young H.A., Wasserman K., Ward J.M., Kleinman H.K., Oppenheim J.J., Murphy W.J. (2000). Human Endothelial Cells Express CCR2 and Respond to MCP-1: Direct Role of MCP-1 in Angiogenesis and Tumor Progression. Blood.

[25] Mulholland B.S., Forwood M.R., Morrison N.A. (2019). Monocyte Chemoattractant Protein-1 (MCP-1/CCL2) Drives Activation of Bone Remodelling and Skeletal Metastasis. Curr. Osteoporos. Rep..

[26] Rajkumar S.V., Dimopoulos M.A., Palumbo A., Blade J., Merlini G., Mateos M.V., Kumar S., Hillengass J., Kastritis E., Richardson P. (2014). International Myeloma Working Group Updated Criteria for the Diagnosis of Multiple Myeloma. Lancet Oncol..

[27] Durie B.G.M., Salmon S.E. (1975). A Clinical Staging System for Multiple Myeloma Correlation of Measured Myeloma Cell Mass with Presenting Clinical Features, Response to Treatment, and Survival. Cancer.

[28] Greipp P.R., Miguel J.S., Dune B.G.M., Crowley J.J., Barlogie B., Bladé J., Boccadoro M., Child J.A., Harousseau J.L., Kyle R.A. (2005). International Staging System for Multiple Myeloma. J. Clin. Oncol..

[29] Oken M.M., Creech R.H., Tormey D.C., Horton J., Davis T.E., McFadden E.T., Carbone P.P. (1982). Toxicity and Response Criteria of the Eastern Cooperative Oncology Group. Am. J. Clin. Oncol..

[30] Terebelo H.R., Reap L., Terebelo H.R., Reap L. (2021). Prognostic and Predictive Factors in Newly Diagnosed Multiple Myeloma Patients with Early Mortality with Prediction Matrix and Three and Five-Year Overall Survival. Multiple Myeloma.

[31] Schaefers C., Seidel C., Bokemeyer F., Bokemeyer C. (2022). The Prognostic Impact of the Smoking Status of Cancer Patients Receiving Systemic Treatment, Radiation Therapy, and Surgery: A Systematic Review and Meta-Analysis. Eur. J. Cancer.

[32] Mansoor W., Roeland E.J., Chaudhry A., Liepa A.M., Wei R., Knoderer H., Abada P., Chatterjee A., Klempner S.J. (2021). Early Weight Loss as a Prognostic Factor in Patients with Advanced Gastric Cancer: Analyses from REGARD, RAINBOW, and RAINFALL Phase III Studies. Oncologist.

[33] Bumma N., Nagasaka M., Hemingway G., Miyashita H., Chowdhury T., Kim S., Vankayala H.M., Ahmed S., Jasti P. (2020). Effect of Exposure to Agent Orange on the Risk of Monoclonal Gammopathy and Subsequent Transformation to Multiple Myeloma: A Single-Center Experience From the Veterans Affairs Hospital, Detroit. Clin. Lymphoma Myeloma Leuk..

[34] Kumar S., Paiva B., Anderson K.C., Durie B., Landgren O., Moreau P., Munshi N., Lonial S., Bladé J., Mateos M.V. (2016). International Myeloma Working Group Consensus Criteria for Response and Minimal Residual Disease Assessment in Multiple Myeloma. Lancet Oncol..

[35] National Cancer Institute (2017). Common Terminology Criteria for Adverse Events (CTCAE) Common Terminology Criteria for Adverse Events (CTCAE) v5.0.

[36] Morgan E., Varro R., Sepulveda H., Ember J.A., Apgar J., Wilson J., Lowe L., Chen R., Shivraj L., Agadir A. (2004). Cytometric Bead Array: A Multiplexed Assay Platform with Applications in Various Areas of Biology. Clin. Immunol..

[37] Kany S., Vollrath J.T., Relja B. (2019). Cytokines in Inflammatory Disease. Int. J. Mol. Sci..

[38] Than S., Hu R., Oyaizu N., Romano J., Wang X.P., Sheikh S., Pahwa S. (1997). Cytokine Pattern in Relation to Disease Progression in Human Immunodeficiency Virus-Infected Children. J. Infect. Dis..

[39] Mehtap O., Atesoglu E.B., Tarkun P., Hacihanefioglu A., Dolasik I., Musul M.M. (2014). IL-21 and Other Serum Proinflammatory Cytokine Levels in Patients with Multiple Myeloma at Diagnosis. J. Postgrad. Med..

[40] Fairfield H., Falank C., Avery L., Reagan M.R. (2016). Multiple Myeloma in the Marrow: Pathogenesis and Treatments. Ann. N. Y. Acad. Sci..

[41] Gernone A., Dammacco F. (1996). Molecular Alterations of IL-6R, Lck and c-Myc Genes in Transforming Monoclonal Gammopathies of Undetermined Significance. Br. J. Haematol..

[42] Lauta V.M. (2003). A Review of the Cytokine Network in Multiple Myeloma: Diagnostic, Prognostic, and Therapeutic Implications. Cancer.

[43] Gadó K., Domján G., Hegyesi H., Falus A. (2000). Role of Interleukin-6 in the Pathogenesis of Multiple Myeloma. Cell Biol. Int..

[44] Gupta V.A., Matulis S.M., Conage-Pough J.E., Nooka A.K., Kaufman J.L., Lonial S., Boise L.H. (2017). Bone Marrow Microenvironment-Derived Signals Induce Mcl-1 Dependence in Multiple Myeloma. Blood.

[45] Li X., Wang J., Zhu S., Zheng J., Xie Y., Jiang H., Guo J., Wang Y., Peng Z., Wang M. (2021). DKK1 Activates Noncanonical NF-ΚB Signaling via IL-6–Induced CKAP4 Receptor in Multiple Myeloma. Blood Adv..

[46] Mondello P., Cuzzocrea S., Navarra M., Mian M. (2017). Bone Marrow Micro-Environment Is a Crucial Player for Myelomagenesis and Disease Progression. Oncotarget.

[47] Kyrtsonis M.C., Dedoussis G., Zervas C., Perifanis V., Baxevanis C., Stamatelou M., Maniatis A. (1996). Soluble Interleukin-6 Receptor (SIL-6R), a New Prognostic Factor in Multiple Myeloma. Br. J. Haematol..

[48] Bataille R., Jourdan M., Zhang X.G., Klein B. (1989). Serum Levels of Interleukin 6, a Potent Myeloma Cell Growth Factor, as a Reflect of Disease Severity in Plasma Cell Dyscrasias. J. Clin. Investig..

[49] Huber H., Krainer M., Herold M., Ludwig H., Wachter H., Huber H. (1991). Predictive Value of Interleukin-6 and Neopterin in Patients with Multiple Myeloma. Cancer Res..

[50] Sfiridaki K., Pappa C.A., Tsirakis G., Kanellou P., Kaparou M., Stratinaki M., Sakellaris G., Kontakis G., Alexandrakis M.G. (2011). Angiogenesis-Related Cytokines, RANKL, and Osteoprotegerin in Multiple Myeloma Patients in Relation to Clinical Features and Response to Treatment. Mediat. Inflamm..

[51] Pedersen B.K., Steensberg A., Schjerling P. (2001). Muscle-Derived Interleukin-6: Possible Biological Effects. J. Physiol..

[52] Febbraio M.A., Pedersen B.K. (2002). Muscle-Derived Interleukin-6: Mechanisms for Activation and Possible Biological Roles. FASEB J..

[53] Pedersen B.K., Steensberg A., Fischer C., Keller C., Keller P., Plomgaard P., Febbraio M., Saltin B. (2003). Searching for the Exercise Factor: Is IL-6 a Candidate?. J. Muscle Res. Cell Motil..

[54] Darko S.N., Yar D.D., Owusu-Dabo E., Awuah A.A.A., Dapaah W., Addofoh N., Salifu S.P., Awua-Boateng N.Y., Adomako-Boateng F. (2015). Variations in Levels of IL-6 and TNF-α in Type 2 Diabetes Mellitus between Rural and Urban Ashanti Region of Ghana. BMC Endocr. Disord..

[55] Sunyer J., Forastiere F., Pekkanen J., Plana E., Kolz M., Pistelli R., Jacquemin B., Brüske-Hohlfeld I., Pitsavos C., Bellander T. (2009). Interaction between Smoking and the Interleukin-6 Gene Affects Systemic Levels of Inflammatory Biomarkers. Nicotine Tob. Res..

[56] Chakraborty B., Vishnoi G., Gowda S.H., Goswami B. (2017). Interleukin-6 Gene-174 G/C Promoter Polymorphism and Its Association with Clinical Profile of Patients with Multiple Myeloma. Asia Pac. J. Clin. Oncol..

[57] Scott H.R., McMillan D.C., Crilly A., McArdle C.S., Milroy R. (1996). The Relationship between Weight Loss and Interleukin 6 in Non-Small-Cell Lung Cancer. Br. J. Cancer.

[58] Fearon K.C.H., Glass D.J., Guttridge D.C. (2012). Cancer Cachexia: Mediators, Signaling, and Metabolic Pathways. Cell Metab..

[59] Moses A.G.W., Maingay J., Sangster K., Fearon K.C.H., Ross J.A. (2009). Pro-Inflammatory Cytokine Release by Peripheral Blood Mononuclear Cells from Patients with Advanced Pancreatic Cancer: Relationship to Acute Phase Response and Survival. Oncol. Rep..

[60] Han J., Meng Q., Shen L., Wu G. (2018). Interleukin-6 Induces Fat Loss in Cancer Cachexia by Promoting White Adipose Tissue Lipolysis and Browning. Lipids Health Dis..

[61] Paval D.R., Patton R., McDonald J., Skipworth R.J.E., Gallagher I.J., Laird B.J. (2022). A Systematic Review Examining the Relationship between Cytokines and Cachexia in Incurable Cancer. J. Cachexia Sarcopenia Muscle.

[62] Liu Y., Zhang Z., Han D., Zhao Y., Yan X., Cui S. (2022). Association between Environmental Chemicals Co-Exposure and Peripheral Blood Immune-Inflammatory Indicators. Front. Public. Health.

[63] Brighton T.A., Khot A., Harrison S.J., Ghez D., Weiss B.M., Kirsch A., Magen H., Gironella M., Oriol A., Streetly M. (2019). Randomized, Double-Blind, Placebo-Controlled, Multicenter Study of Siltuximab in High-Risk Smoldering Multiple Myeloma. Clin. Cancer Res..

[64] Orlowski R.Z., Gercheva L., Williams C., Sutherland H., Robak T., Masszi T., Goranova-Marinova V., Dimopoulos M.A., Cavenagh J.D., Špička I. (2015). A Phase 2, Randomized, Double-Blind, Placebo-Controlled Study of Siltuximab (Anti-IL-6 MAb) and Bortezomib versus Bortezomib Alone in Patients with Relapsed or Refractory Multiple Myeloma. Am. J. Hematol..

[65] Harmer D., Falank C., Reagan M.R. (2019). Interleukin-6 Interweaves the Bone Marrow Microenvironment, Bone Loss, and Multiple Myeloma. Front. Endocrinol..

[66] Shapiro V.S., Mollenauer M.N., Weiss A. (2001). Endogenous CD28 Expressed on Myeloma Cells Up-Regulates Interleukin-8 Production: Implications for Multiple Myeloma Progression. Blood.

[67] Pellegrino A., Ria R., Di Pietro G., Cirulli T., Surico G., Pennisi A., Morabito F., Ribatti D., Vacca A. (2005). Bone Marrow Endothelial Cells in Multiple Myeloma Secrete CXC-Chemokines That Mediate Interactions with Plasma Cells. Br. J. Haematol..

[68] Wyczalkowska-Tomasik A., Czarkowska-Paczek B., Zielenkiewicz M., Paczek L. (2016). Inflammatory Markers Change with Age, but Do Not Fall Beyond Reported Normal Ranges. Arch. Immunol. Ther. Exp..

[69] Clark J.A., Peterson T.C. (1994). Cytokine Production and Aging: Overproduction of IL-8 in Elderly Males in Response to Lipopolysaccharide. Mech. Ageing Dev..

[70] Liu K.D., Altmann C., Smits G., Krawczeski C.D., Edelstein C.L., Devarajan P., Faubel S. (2009). Serum Interleukin-6 and Interleukin-8 Are Early Biomarkers of Acute Kidney Injury and Predict Prolonged Mechanical Ventilation in Children Undergoing Cardiac Surgery: A Case-Control Study. Crit. Care.

[71] Tunçay S.C., Doğan E., Hakverdi G., Tutar Z.Ü., Mir S. (2021). Interleukin-8 Is Increased in Chronic Kidney Disease in Children, but not Related to Cardiovascular Disease. J. Bras. Nefrol..

[72] Polańska B., Augustyniak D., Makulska I., Niemczuk M., Jankowski A., Zwolińska D. (2014). Elastase, A1-Proteinase Inhibitor, and Interleukin-8 in Children and Young Adults with End-Stage Kidney Disease Undergoing Continuous Ambulatory Peritoneal Dialysis. Arch. Immunol. Ther. Exp..

[73] Liu S.-Y., Chen J., Li Y.-F. (2018). Clinical Significance of Serum Interleukin-8 and Soluble Tumor Necrosis Factor-like Weak Inducer of Apoptosis Levels in Patients with Diabetic Nephropathy. J. Diabetes Investig..

[74] D’agostino M., Cairns D.A., Lahuerta J.J., Wester R., Bertsch U., Waage A., Zamagni E., Mateos M.V., Dall’olio D., Van De Donk N.W.C.J. (2022). Second Revision of the International Staging System (R2-ISS) for Overall Survival in Multiple Myeloma: A European Myeloma Network (EMN) Report Within the HARMONY Project. J. Clin. Oncol..

[75] Ikezumi Y., Uemura O., Nagai T., Ishikura K., Ito S., Hataya H., Fujita N., Akioka Y., Kaneko T., Iijima K. (2015). Beta-2 Microglobulin-Based Equation for Estimating Glomerular Filtration Rates in Japanese Children and Adolescents. Clin. Exp. Nephrol..

[76] Bianchi C., Donadio C., Tramonti G., Consani C., Lorusso P., Rossi G. (2001). Reappraisal of Serum Beta2-Microglobulin as Marker of GFR. Ren. Fail..

[77] Assounga A.G. (2021). Beta 2 Microglobulin in Kidney Failure: A Review and an Algorithm for Renal Replacement Therapy. Saudi J. Kidney Dis. Transplant..

[78] Sedighi O., Abediankenari S., Omranifar B. (2015). Association Between Plasma Beta-2 Microglobulin Level and Cardiac Performance in Patients with Chronic Kidney Disease. Nephrourol. Mon..

[79] Tripathy N.K., Vibhuti, Nityanand S. (2005). Bone Marrow and Blood Plasma Levels of IL-8 in Aplastic Anemia and Their Relationship with Disease Severity. Am. J. Hematol..

[80] Saltarella I., Morabito F., Giuliani N., Terragna C., Omedè P., Palumbo A., Bringhen S., De Paoli L., Martino E., Larocca A. (2019). Prognostic or Predictive Value of Circulating Cytokines and Angiogenic Factors for Initial Treatment of Multiple Myeloma in the GIMEMA MM0305 Randomized Controlled Trial. J. Hematol. Oncol..

[81] Ribas C., Colleoni G.W.B., Regis Silva M.R., Carregoza M.J., Bordin J.O. (2004). Prognostic Significance of Vascular Endothelial Growth Factor Immunoexpression in the Context of Adverse Standard Prognostic Factors in Multiple Myeloma. Eur. J. Haematol..

[82] Palta A., Kaur M., Tahlan A., Dimri K. (2020). Evaluation of Angiogenesis in Multiple Myeloma by VEGF Immunoexpression and Microvessel Density. J. Lab. Physicians.

[83] Di Raimondo F., Azzaro M.P., Palumbo G., Bagnato S., Giustolisi G., Florida P.M., Sortino G., Giustolisi R. (2000). Angiogenic factors in multiple myeloma: Higher levels in bone marrow than in peripheral blood. Haematologica.

[84] Podar K., Tonon G., Sattler M., Tai Y.T., LeGouill S., Yasui H., Ishitsuka K., Kumar S., Kumar R., Pandite L.N. (2006). The Small-Molecule VEGF Receptor Inhibitor Pazopanib (GW786034B) Targets Both Tumor and Endothelial Cells in Multiple Myeloma. Proc. Natl. Acad. Sci. USA.

[85] Zangari M., Anaissie E., Stopeck A., Morimoto A., Tan N., Lancet J., Cooper M., Hannah A., Garcia-Manero G., Faderl S. (2004). Phase II Study of SU5416, a Small Molecule Vascular Endothelial Growth Factor Tyrosine Kinase Receptor Inhibitor, in Patients with Refractory Multiple Myeloma. Clin. Cancer Res..

[86] Prince H.M., Hönemann D., Spencer A., Rizzieri D.A., Stadtmauer E.A., Roberts A.W., Bahlis N., Tricot G., Bell B., DeMarini D.J. (2009). Vascular Endothelial Growth Factor Inhibition Is Not an Effective Therapeutic Strategy for Relapsed or Refractory Multiple Myeloma: A Phase 2 Study of Pazopanib (GW786034). Blood.

[87] Kovacs M.J., Reece D.E., Marcellus D., Meyer R.M., Mathews S., Dong R.P., Eisenhauer E. (2006). A Phase II Study of ZD6474 (Zactima^TM^), a Selective Inhibitor of VEGFR and EGFR Tyrosine Kinase in Patients with Relapsed Multiple Myeloma—NCIC CTG IND.145. Investig. New Drugs.

[88] Valković T., Babarović E., Lučin K., Štifter S., Aralica M., Seili-Bekafigo I., Duletić-Načinović A., Jonjić N. (2016). Plasma Levels of Monocyte Chemotactic Protein-1 Are Associated with Clinical Features and Angiogenesis in Patients with Multiple Myeloma. Biomed. Res. Int..

[89] Liu Z., Xu J., Li H., Zheng Y., He J., Liu H., Zhong Y., Lu Y., Hong B., Zhang M. (2013). Bone Marrow Stromal Cells Derived MCP-1 Reverses the Inhibitory Effects of Multiple Myeloma Cells on Osteoclastogenesis by Upregulating the RANK Expression. PLoS ONE.

[90] Bird S., Cairns D., Menzies T., Boyd K., Davies F., Cook G., Drayson M., Gregory W., Jenner M., Jones J. (2021). Sex Differences in Multiple Myeloma Biology but Not Clinical Outcomes: Results from 3894 Patients in the Myeloma XI Trial. Clin. Lymphoma Myeloma Leuk..

[91] Cook M.B., McGlynn K.A., Devesa S.S., Freedman N.D., Anderson W.F. (2011). Sex Disparities in Cancer Mortality and Survival. Cancer Epidemiol. Biomarkers Prev..

[92] Cavo M., Gay F., Beksac M., Pantani L., Petrucci M.T., Dimopoulos M.A., Dozza L., van der Holt B., Zweegman S., Oliva S. (2020). Autologous Haematopoietic Stem-Cell Transplantation versus Bortezomib-Melphalan-Prednisone, with or without Bortezomib-Lenalidomide-Dexamethasone Consolidation Therapy, and Lenalidomide Maintenance for Newly Diagnosed Multiple Myeloma (EMN02/HO95): A Multicentre, Randomised, Open-Label, Phase 3 Study. Lancet Haematol..

[93] Corre J., Perrot A., Caillot D., Belhadj K., Hulin C., Leleu X., Mohty M., Facon T., Buisson L., Do Souto L. (2021). Del(17p) without TP53 Mutation Confers a Poor Prognosis in Intensively Treated Newly Diagnosed Patients with Multiple Myeloma. Blood.

[94] Rajkumar S.V. (2022). Multiple Myeloma: 2022 Update on Diagnosis, Risk-Stratification and Management. Am. J. Hematol..

