# Supplementary material for: The Clinical Relevance of Selected Cytokines in Newly Diagnosed Multiple Myeloma Patients
Source: Biomedicines. 2023 Nov 9;11(11):3012. doi: 10.3390/biomedicines11113012 (PMC10669681; doi:10.3390/biomedicines11113012)
Supplement: Supplementary file 1 [file biomedicines-11-03012-s001.zip › biomedicines-2630259-supplementary.pdf]

Supplementary Material:

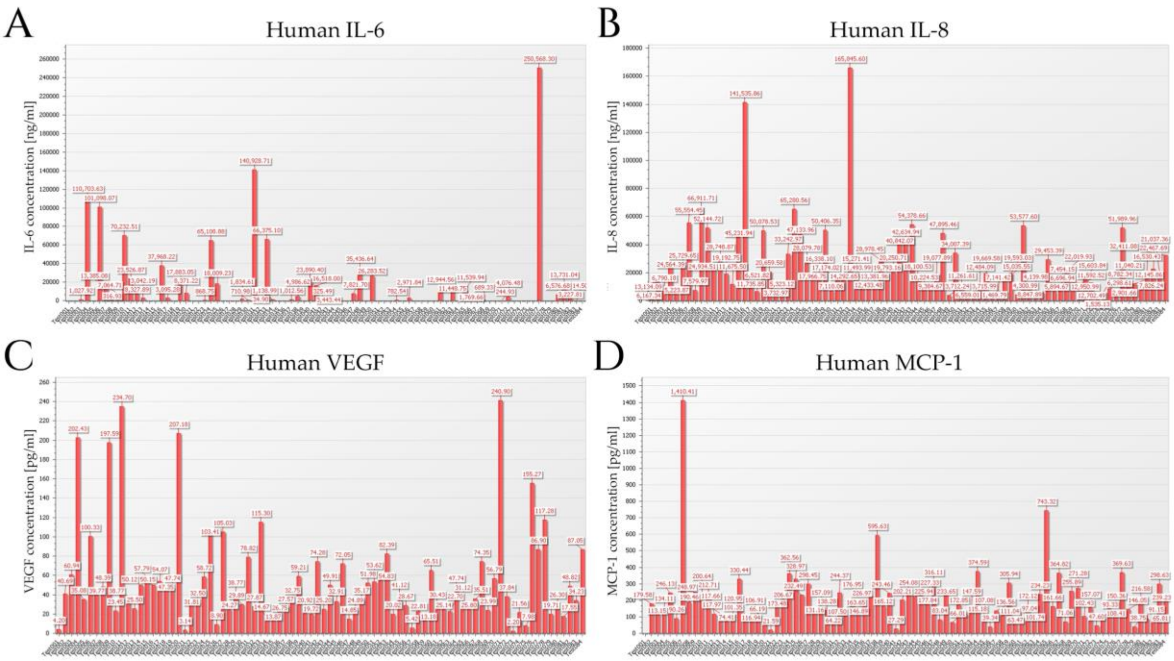

Figure S1. Concentrations of IL-6 (A), IL-8 (B), VEGF (C), and MCP-1 (D) using FCAP Array™ software (Becton Dickinson, New Jersey, USA). Abbreviations: IL-6—interleukin-6; IL-8—interleukin-8; VEGF—vascular endothelial growth factor; MCP-1—angiogenic chemokine monocyte chemoattractant protein-1.

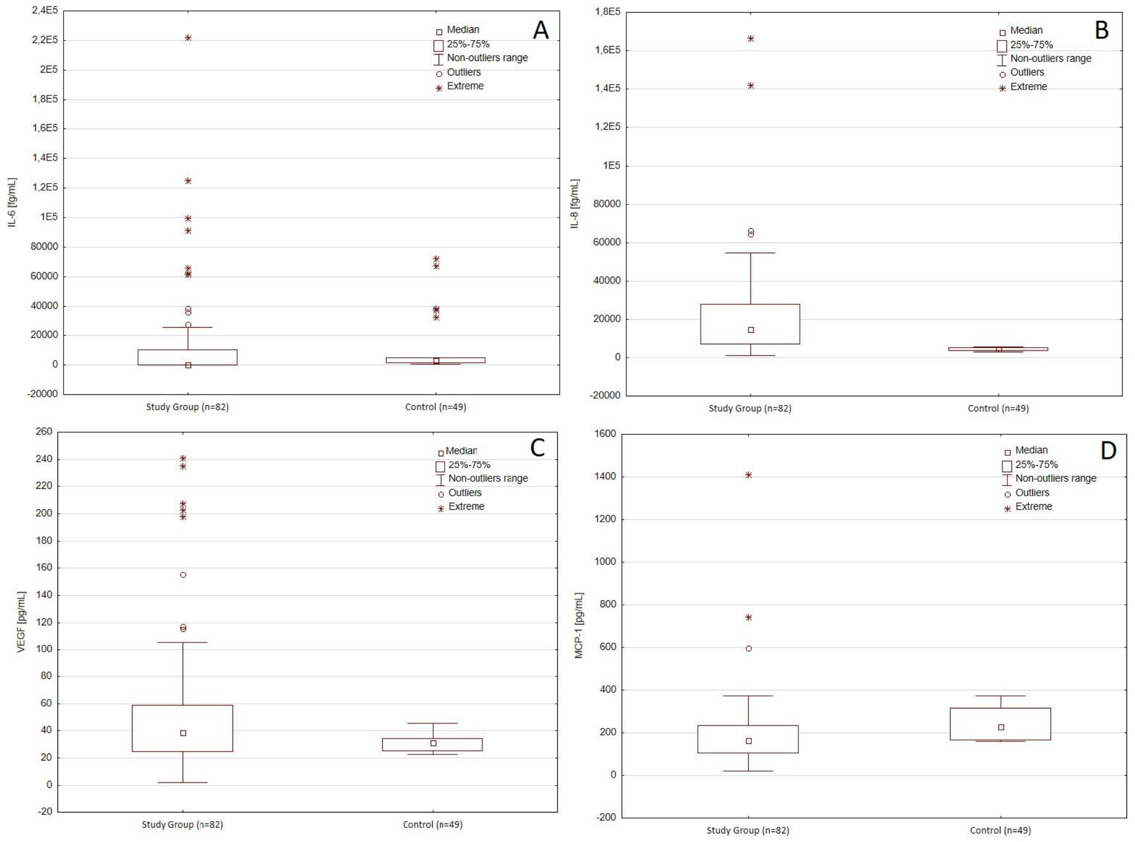

Figure S2. Box-and-whisker plot comparing the concentrations of IL-6 (A), IL-8 (B), VEGF (C), and MCP-1 (D) between the study and control groups. Abbreviations: IL-6—interleukin-6; IL-8—interleukin-8; VEGF—vascular endothelial growth factor; MCP-1—angiogenic chemokine monocyte chemoattractant protein-1.

Table S1. Relationship between demographic and clinical variables and progression-free survival or overall survival in the study group.

Variable	n	Progression-free survival			Overall survival		
		Univariate		Multivariate	Univariate		Multivariate
		Median (months)	HR (95% CI) *p*	HR (95% CI) *p*	Median (months)	HR (95% CI) *p*	HR (95% CI) *p*
**Sex**			1.31 (0.80-2.13) 0.268	0.99 (0.52-1.88) 0.002*		2.17 (1.21-3.88) 0.008*	3.19 (1.17-8.69) 0.002*
Men	40 (48.8%)	25			38		
Women	42 (51.2%)	25			78		
**Age >65 years**			0.61 (0.37-1.01) 0.056	0.31 (0.73-2.65) 0.317		0.65 (0.36-1.17) 0.156	1.36 (0.70-2.65) 0.356
Below the median	40 (48.8%)	27			77		
Above the median	42 (51.2%)	21			55		
**Diagnosis**			0.67 (0.28-1.58) 0.362	1.36 (0.52-3.52) 0.526		0.47 (0.15-1.40) 0.177	2.57 (0.95-6.94) 0.063
Disease with monoclonal protein present	72 (87.8%)	25			57		
Light chain disease	10 (12.2%)	14.5			12		
**A type of monoclonal protein**	20 (27.4%)	18	1.08 (0.59-1.99)	0.99 (0.39-2.47) 0.990	38	1.60 (0.77-3.32)	0.64 (0.31-1.31) 0.227
	53 (72.6%)	25			77		

IgA<br>IgG			0.794			0.208	
**Light chains**							
Kappa	48 (58.5%)	28	0.72 (0.43-1.21)	1.39 (0.75-2.59)	64	0.70 (0.38-1.28)	1.25 (0.68-2.28)
Lambda	34 (41.5%)	17	0.223	0.294	38	0.252	0.470
**ISS Stage**							
2 or 3	58 (71.6%)	17	2.92 (1.77-4.81)	2.38 (0.97-5.86)	48	2.99 (1.62-5.52)	1.89 (0.78-4.54)
1	23 (28.4%)	52	<0.001*	0.059*	-	0.004*	0.157
**A/B renal function**							
B	67 (81.7%)	15	2.12 (0.98-4.55)	2.29 (0.99-5.27)	18	2.78 (1.11-6.96)	1.14 (0.54-2.41)
A	15 (18.3%)	28	0.009*	0.051*	68	0.001*	0.732
**Weight loss**							
Yes	41 (50.6%)	18	1.41 (0.86-2.31)	1.03 (0.73-1.45)	33	2.11 (1.16-3.84)	1.16 (0.81-1.67)
No	40 (49.4%)	34	0.162	0.836	78	0.009*	0.401
**ECOG scale**							
0	8 (9.8%)	24	0.58 (0.26-1.28)	0.54 (0.15-1.95)	124	0.45 (0.18-1.10)	3.39 (0.80-14.32)
1. 2. 3	74 (90.2%)	25	0.184	0.352	55	0.082	0.091
**17p deletion**							
Yes	9 (16.7%)	11	2.72 (0.95-7.78)	2.44 (1.03-5.76)	28	2.83 (0.76-10.42)	2.17 (0.77-6.08)
No	45 (83.3%)	37	0.004*	0.042*	80	0.018*	0.142
**Translocation (4;14)**							
Yes	6 (11.1%)	21	1.05 (0.40-2.78)	1.24 (0.48-3.21)	49	2.38 (0.59-10.00)	2.53 (0.78-8.13)
No	48 (88.9%)	27	0.924	0.655	80	0.227	0.120
**Translocation (14;16)**							
Yes	3 (5.6%)	66	0.61 (0.19-1.92)	0.74 (0.09-5.89)	-	0.71 (0.13-3.92)	0.82 (0.11-6.24)
No	51 (94.4%)	27	0.487	0.779	77	0.730	0.852
**Auto-HSCT**							
No	45 (59.2%)	13	2.61 (1.56-4.38)	0.66 (0.31-1.42)	30	2.79 (1.53-5.08)	0.43 (0.16-1.09)
Yes	31 (40.8%)	39	<0.001*	0.297	-	<0.001*	0.08*
**Anemia before treatment (WHO)**							
No	21 (25.6%)	34	0.56 (0.33-0.95)	1.34 (0.63-2.81)	57	0.79 [0.42-1.47]	1.70 (0.88-3.30)
Yes	61 (74.4%)	18	0.033	0.438	55	0.457	0.115
**IL-6 (M 0.1)**							
High	40 (49.4%)	17	1.89 [1.15-3.11]	1.19 (0.60-2.35)	28	2.62 [1.45-4.70]	3.30 (1.24-8.76)
Low	41 (50.6%)	35	0.008*	0.605	78	<0.001*	0.017*
**IL-8 (M 14823.57)**							
High	40 (49.4%)	17	1.65 [1.01-2.71]	0.84 (0.41-1.70)	38	1.70 [0.94-3.07]	1.22 (0.64-2.33)
Low	41 (50.6%)	28	0.037*	0.631	68	0.062	0.532

**VEGF (M 38.77)**							
High	39 (48.1%)	23	1.57 [0.95-2.58]	0.80 (0.41-1.57)	51	1.34 [0.74-2.41]	1.38 (0.76-2.50)
Low	42 (51.9%)	27	0.060	0.531	57	0.305	0.291
**MCP-1 (M 161.66)**							
High	40 (49.4%)	25	0.79 [0.48-1.28]	1.26 (0.61-2.56)	57	0.80 [0.45-1.43]	0.76 (0.41-1.40)
Low	41 (50.6%)	25	0.337	0.526	55	0.448	0.393

* Statistically significant result.

Table S2. Evaluation of the usefulness of IL-6, IL-8, and VEGF concentrations in differentiating between various clinical conditions.

			**IL-6**			
**Variable**		**AUC [95% CI]**	**Sensitivity %**	**Specificity %**	**Cut-off point**	***p***
**ISS stage**						
I	23 (28.4%)	0.64 [0.53-0.75]	50.00	81.82	>1165.53	0.011*
II or III	58 (71.6%)					
**Weight loss before treatment**						
No	41 (50.6%)	0.70 [0.59-0.80]	67.50	77.50	>756.68	<0.001*
Yes	40 (49.4%)					
			**IL-8**			
**Variable**		**AUC [95% CI]**	**Sensitivity %**	**Specificity %**	**Cut-off point**	***p***
**ISS stage**						
I or II	23 (28.4%)	0.65 [0.54-0.75]	55.17	72.73	>15546.65	0.022*
III	58 (71.6%)					
**A/B renal function**						
A	67 (81.7%)	0.84 [0.74-0.91]	80.00	77.27	>20191	<0.001*
B	15 (18.3%)					
**ECOG scale**						
0	8 (9.8%)	0.75 [0.65-0.84]	75.68	85.71	>8738.92	0.010*
1, 2, or 3	74 (90.2%)					
**Supportive treatment**						
No	21 (25.6%)	0.77 [0.66-0.85]	65.57	80.00	≤16370.78	<0.001*
Yes	61 (74.4%)					
**Auto-HSCT**						
No	45 (59.2%)	0.68 [0.56-0.78]	64.52	65.91	≤14264.49	0.003*
Yes	31 (40.8%)					
**Anemia before treatment**						
No	21 (25.6%)	0.79 [0.68-0.87]	76.67	80.95	>11039.5	<0.001*
Yes	61 (74.4%)					
			VEGF			
**Variable**		**AUC [95% CI]**	**Sensitivity %**	**Specificity %**	**Cut-off point**	***p***
**A/B renal function**						
A	67 (81.7%)	0.73 [0.62-0.82]	93.33	59.09	>37.84	<0.001*
B	15 (18.3%)					

* Statistically significant result.

Table S3. Comparison of the baseline concentrations of tested cytokines depending on the response to CTH after four, six, and eight cycles.

Variable	Study group (n=82)	IL-6 [fg/mL] Median [interquartile range]	IL-6 *p*	IL-8 [fg/mL] Median [interquartile range]	IL-8 *p*	VEGF [pg/mL] Median [interquartile range]	VEGF *p*	MCP-1 [pg/mL] Median [interquartile range]	MCP-1 *p*
**Response to CTH after four cycles**									
No	4 (6.2%)	14632.96 [–]		21212.48 [11595.20-37613.07]		59.35 [28.65-160.80]		193.09 [145.72-291.17]	
Yes	60 (93.7%)	0.10 [0.10-4200.37]	0.085	12397.80 [6546.33-19715.46]	0.310	35.08 [24.48-58.11]	0.382	157.07 [101.91-231.20]	0.310
**Response to CTH after six cycles**									
No	2 (3.4%)	110868.48 [–]		29660.28 [–]		47.44 [–]		259.99 [–]	
Yes	56 (96.6%)	0.10 [0.10-4200.37]	0.463	13534.43 [6546.33-20097.42]	0.487	35.08 [25.30-58.11]	0.828	161.66 [103.59-234.08]	0.340
**Response to CTH after eight cycles**									
No	4 (16.7%)	0.10 [0.10-4155.35]		9562.55 [7692.04-14127.92]		38.10 [17.14-50.01]		183.47 [112.38-221.26]	
Yes	20 (83.3%)	0.10 [0.10-4653.81]	0.583	11843.35 [6340.05-18897]	0.877	29.28 [14.36-36.67]	0.757	159.36 [107.74-271.50]	0.938

* Statistically significant result.
